# Personalized prediction for multiple chronic diseases by developing the multi-task Cox learning model

**DOI:** 10.1371/journal.pcbi.1011396

**Published:** 2023-09-21

**Authors:** Shuaijie Zhang, Fan Yang, Lijie Wang, Shucheng Si, Jianmei Zhang, Fuzhong Xue

**Affiliations:** 1 Department of Epidemiology and Health Statistics, School of Public Health, Cheeloo College of Medicine, Shandong University, Jinan, China; 2 National Institute of Health Data Science of China; 3 Department of Geriatrics, Weihai Municipal Hospital Affiliated Shandong University, 76 Heping Rd, Weihai, Shandong, China; Washington State University, UNITED STATES

## Abstract

Personalized prediction of chronic diseases is crucial for reducing the disease burden. However, previous studies on chronic diseases have not adequately considered the relationship between chronic diseases. To explore the patient-wise risk of multiple chronic diseases, we developed a multitask learning Cox (MTL-Cox) model for personalized prediction of nine typical chronic diseases on the UK Biobank dataset. MTL-Cox employs a multitask learning framework to train semiparametric multivariable Cox models. To comprehensively estimate the performance of the MTL-Cox model, we measured it via five commonly used survival analysis metrics: concordance index, area under the curve (AUC), specificity, sensitivity, and Youden index. In addition, we verified the validity of the MTL-Cox model framework in the Weihai physical examination dataset, from Shandong province, China. The MTL-Cox model achieved a statistically significant (p<0.05) improvement in results compared with competing methods in the evaluation metrics of the concordance index, AUC, sensitivity, and Youden index using the paired-sample Wilcoxon signed-rank test. In particular, the MTL-Cox model improved prediction accuracy by up to 12% compared to other models. We also applied the MTL-Cox model to rank the absolute risk of nine chronic diseases in patients on the UK Biobank dataset. This was the first known study to use the multitask learning-based Cox model to predict the personalized risk of the nine chronic diseases. The study can contribute to early screening, personalized risk ranking, and diagnosing of chronic diseases.

## Author summary

Chronic diseases present a significant challenge to public healthcare systems, responsible for almost 60% of global fatalities. Addressing this issue requires the implementation of preventative treatments and interventions to enable the early detection of chronic diseases in those who are at risk. However, most research in the field of chronic disease risk prediction has focused only on single-disease prediction tasks, without adequate attention paid to the connections between chronic diseases. Thus, a multitask learning Cox model was developed to predict nine typical chronic diseases (lung cancer, gastric cancer, esophagus cancer, colorectal cancer, liver cancer, hypertension, diabetes, stroke, and coronary heart disease), based on data from the UK Biobank. In addition, the validity of the MTL-Cox model framework was also demonstrated with the Weihai physical examination dataset in China. Our experimental results indicate that the MTL-Cox model could offer personalized absolute risk prediction for chronic diseases.

## Introduction

According to a commonly accepted definition, chronic diseases are conditions that last for at least one year and require ongoing medical attention or limit daily activities [1], which are a leading cause of death and disability worldwide [2]. In the United States, chronic diseases account for approximately 84% of the nation’s healthcare spending, which totals $3.8 trillion annually (Anderson G. Chronic Care: Making the Case for Ongoing Care. Princeton, NJ: Robert Wood Johnson Foundation, 2010. Available at: http://www.rwjf.org/content/dam/farm/reports/reports/2010/rwjf54583. Accessed on Apr. 02^*nd*^, 2022.). Furthermore, chronic diseases such as stroke, diabetes, cancer, and cardiovascular diseases like hypertension and *c*oronary *h*eart *d*isease (CHD) account for nearly 60% of global fatalities [3–5]. Lung cancer, gastric cancer, liver cancer, colorectal cancer, and esophageal cancer have high morbidity rates among both males and females [6]. As a result, this study aims to research the nine chronic diseases mentioned above.

Clinical personalized risk prediction algorithms are powerful tools for healthcare management [7]. These algorithms assist clinicians in developing effective intervention strategies for patients and optimize the allocation of medical resources. In view of their significance, numerous machine learning methods have been developed for estimating the personalized risk of patients [8–12]. However, developing clinical personalized risk prediction models is challenging due to the uncertain follow-up time of subjects. Given time and cost constraints, it is difficult to track all subjects until an event of interest occurs. In other words, not all subjects experience an event of interest during the follow-up period, and some drop out for various reasons, making it impossible to observe the event of interest. However, this does not imply that such events will never occur in the future. In statistics, these drop-out subjects are referred to as *right-censored* [13, 14], accounting for a large proportion of real-world health data [15], leading to bias in survival analysis [16]. Therefore, it is essential to consider right-censored subjects while predicting disease risk. This paper addresses the problem of right-censoring by utilizing the Cox proportional hazards model.

Chronic diseases, such as cardiovascular diseases, cancer, chronic respiratory diseases, and diabetes, are closely linked from a physiological perspective (www.cdc.gov/chronicdisease/about/index.htm. Accessed on Sep. 17^*th*^, 2022.) [17]. Based on this insight, some epidemiological evidence suggested that there exist common risk factors causing chronic diseases [18, 19]. It is worth noting that one chronic disease can often be accompanied by others, which means that the correlation between these diseases can be used to prevent additional chronic diseases. However, most studies on chronic disease prevention use single-task learning-based methods that only focus on capturing individual risk probabilities, without considering the interplay between multiple diseases [20–26]. This approach often results in weak model generalization and limited ability to capture multiple diseases. To address these challenges, this study employs a *m*ulti*t*ask *l*earning (MTL) framework that leverages the correlations among multiple diseases to improve model performance and generalization. The MTL framework, originally proposed by Caruana [27], considers two tasks to be related if they share the same feature space during model training. By learning multiple related tasks together, models can be optimized for improved performance [28].

**Fig 1:**
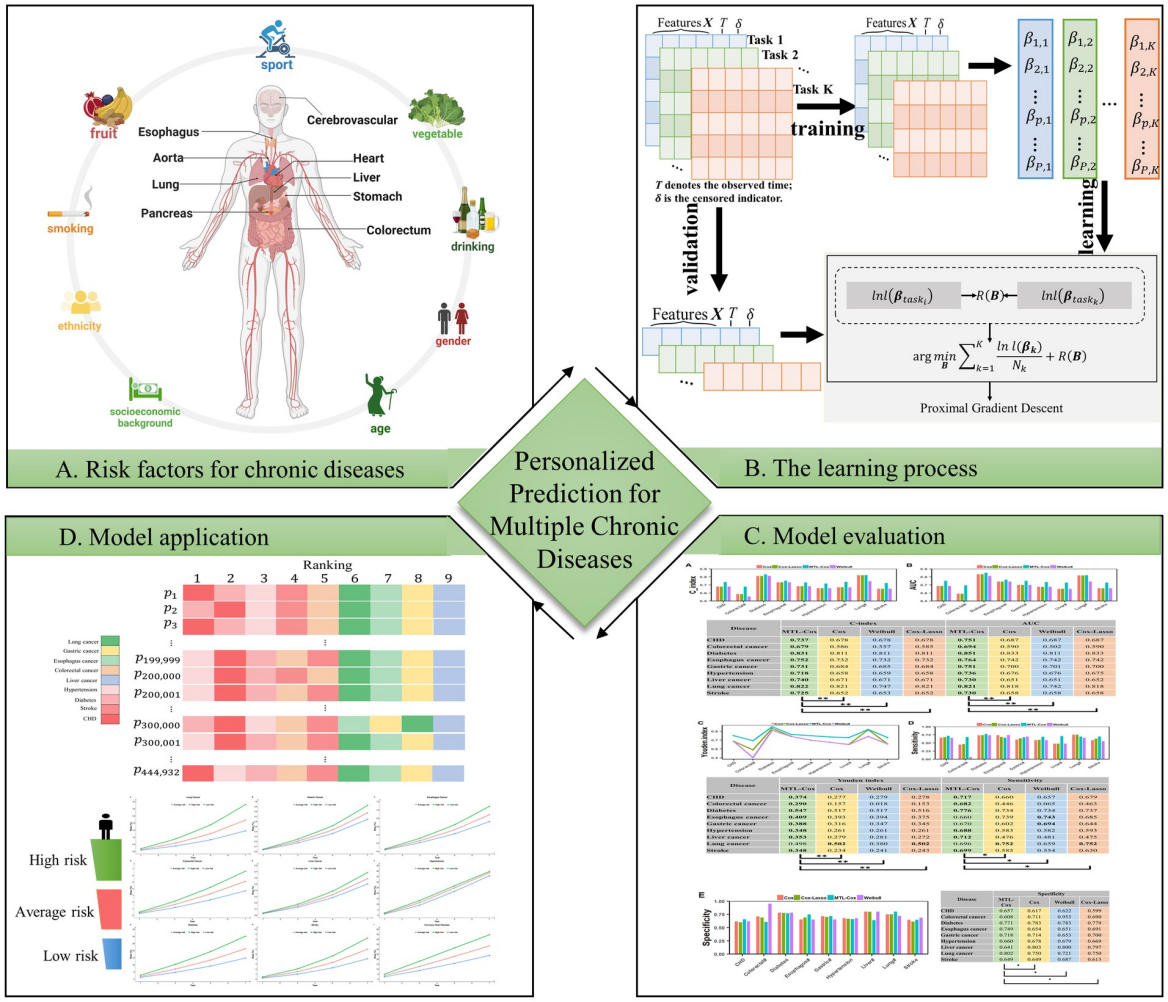
The framework of the MTL-Cox model. (A) The inner circle shows the main sites of the nine chronic diseases, and the outer circle demonstrates factors that affect chronic diseases in the UK Biobank data, which is detailed in the Materials Section. The figure is created with *Biorender*. (B) The flow of the MTL-Cox model construction and optimization, which detailed in the Model Section; (C) Concordance index, AUC, specificity, sensitivity, and Youden index are used to evaluate models, which detailed in the Experiments and results Section; (D) Applying the MTL-Cox model for chronic diseases personalized prediction, which detailed in the Experiments and results Section.

In this paper, we address the problem of predicting multiple chronic diseases using right-censored data by proposing an MTL-based survival analysis method called MTL-Cox, which trains semi-parametric *Cox* proportional hazards models via a *m*ulti*t*ask *l*earning framework. By sharing latent representations across feature spaces, MTL enables multiple tasks related to chronic disease risk prediction to be learned in parallel. To ensure the model’s robustness, we update MTL-Cox by adding the *L*_2,1_ norm as a regularization term to promote similar parameter sparsity patterns among multiple chronic disease predictors. Additionally, we employ the proximal gradient method to train MTL-Cox to converge at a fast rate of O(1/ε). To evaluate the proposed framework, we demonstrate its risk prediction performance using demographic and clinical data from the UK Biobank and verify the validity of the MTL-Cox model framework on the Weihai physical examination dataset. The experimental results show that our proposed framework outperformed competing models, and Fig 1 conveys the big picture and flow process of MTL-Cox.

Our contributions are as follows:

1. Proposed an MTL-based survival analysis method called MTL-Cox for predicting multiple chronic diseases using right-censored data. Enabled multiple tasks related to chronic disease risk prediction to be learned in parallel by sharing latent representations across feature spaces via MTL.
2. Demonstrated the superior risk prediction performance of the proposed framework using demographic and clinical data from the UK Biobank and validated the MTL-Cox model framework on the Weihai physical examination dataset.
3. Defined five measurements to identify the personalized risk.

The rest of the paper is structured as follows. The Related Works section presents the existing literature on disease risk signaling using statistical and machine learning methods. The Notations and Preliminaries section introduces the notations used in this study and provides a basic background. The Model section provides a detailed explanation of the MTL-Cox framework. In the Materials section, we showcase data from the UK Biobank and the Weihai physical examination dataset, along with details of data processing. The Experiments and Results section demonstrates the multiple chronic disease risk prediction for patients and diseases separately. The Discussion section quantitatively evaluates the performance of the proposed model using five measurement metrics, including the concordance index, AUC, specificity, sensitivity, and Youden index. We summarize the essential findings in the Conclusion section. Lastly, the Future Work section discusses potential strategies for improving MTL-Cox.

## Related works

Extensive literature has been devoted to the analysis of chronic disease survival analysis in the past few decades. The approaches employed in these studies can be broadly categorized into two groups: statistical methods and machine learning techniques.

### Statistical methods

In this section, we present various statistical methods utilized for survival analysis, namely nonparametric, semi-parametric, and parametric methods.

Nonparametric methods, such as the Kaplan-Meier [29] and Life-Table methods [30], are typically employed when the data distribution is unavailable. However, these methods fail to capture individual differences in disease risk perception. Parametric methods, on the other hand, rely on a specific data distribution and are thus limited in their ability to adapt to diverse time-to-event samples for disease risk analysis [31, 32]. In the semi-parametric category, the Cox proportional hazards model is the most commonly used for survival analysis [33]. This method overcomes the shortcomings of both nonparametric and parametric methods by taking individual differences into account, and providing personalized predictions for each subject. Furthermore, the parameter estimation does not require survival times to follow a specific distribution. Therefore, we have employed the Cox model for personalized prediction of chronic disease survival.

### Machine learning methods

Over the past few decades, the computing community has made significant contributions to the field of survival analysis for chronic diseases. The research in this area can be broadly classified into single- and multiple-disease prediction.

For single-disease prediction, various methods have been proposed by different researchers, such as Jiang et al. [34] who developed *s*upport *v*ector *m*achine (SVM)-based classification methods to identify gastric cancer, which showed promising results. Kumardeep et al. [35] formulated a robust survival analysis model to predict liver cancer by using autoencoder and SVM techniques. Siavash and Mohammad [36] proposed a novel decision tree-based method to identify risk factors of esophagus cancer and predict early readmission following esophagus cancer. Among the models proposed, some belong to a neural network, such as the knowledge-guided convolutional neural network model developed by Ye et al. [37] to predict the risk of mortality in critically ill patients with diabetes, which yielded a competitive AUC performance. Additionally, a Bayesian neural network was proposed to predict the survival of gastric cancer patients [38]. As AI technology evolves, researchers have started using ensemble learning methods and deep learning models for disease prediction, such as Diao et al. [39] adoption of E*x*treme *G*radient *Boost*ing (XGBoost) to predict common etiologies in patients with suspected secondary hypertension, and She et al. [40] use of the *deep* learning *surv*ival neural network model (DeepSurv) model to predict non-small cell lung cancer, which provided desirable prediction accuracy and individual survival information. However, these methods can only predict one disease at a time.

For multiple-disease prediction, unlike the methods mentioned above, an advanced machine learning technique called multitask learning has been developed to create an integrated multiple-disease predictor through parallel learning. In 2021, Feng et al. [41] proposed an MTL-based framework for predicting hypertension and type-2 diabetes, where a two-branch network was developed for each disease. Each branch consisted of two convolutional layers and two fully connected layers. The results demonstrated that the MTL model achieved better performance than single-task models. Additionally, Anusha et al. [42] utilized the deep belief network and recurrent neural network for multi-disease prediction, which showed superior performance. However, these models do not consider the right censoring.

The studies mentioned above have demonstrated promising results in disease prediction. However, there is still potential for further improvement. Most of the relevant methods only focus on predicting survival rates for single diseases and do not consider the correlation between chronic diseases. Additionally, while some studies have proposed models for predicting multiple diseases, these models have limitations in handling right-censored data. It is essential to address these issues from a perspective of robustness and generalization for multiple-disease survival analysis. In this study, we propose the use of semi-parametric Cox models for multiple diseases in the multitask learning framework. The MTL-Cox model is specifically designed for right-censored data and achieves excellent performance by simultaneously learning multiple related tasks.

## Notations and preliminaries

The notations and preliminary definitions used in this study that has been presented in this section.

### Notations

All vectors are denoted by bold lowercase letters (e.g., ***x***_*i*_), while matrices are represented by bold uppercase letters (e.g., ***X***). A summary of the notations used in this paper is presented in Table 1.

**Table 1:** Notations of symbols that used in this paper.

Notation	Description
*N*	Number of subjects.
*P*	Number of features.
*K*	Number of tasks.
*T*	Survival/failure time, where the dimension is $R^{N\times 1}$.
*δ*	Binary vector for event status, where the dimension is *N* × 1.
*R*(*T*_*i*_)	The risk set at *T*_*i*_.
***X***	The feature matrix, where the dimension is $R^{N\times P}$.
***x***_*p*_	The feature vector of *p*-*th* feature for *N* subjects, where the dimension is $R^{N\times 1}$.
***β***	The coefficient vector for single task learning, where the dimension is $R^{P\times 1}$.
*β*_*p*_	The coefficient of *p*-*th* feature for single-task learning.
***β***_*k*_	The coefficient vector of *k*-*th* task’s features for multitask learning, where the dimension is $R^{P\times 1}$.
***B***	The coefficients matrix of all tasks’ features, where the dimension is $R^{P\times K}$.

### Preliminaries

#### Cox proportional hazards model

The Cox model is a widely used method in survival analysis that effectively utilizes survival or failure time (*T*_*i*_). A complete survival dataset consists of three elements: start time, end time, and end event. In this study on the UK Biobank dataset, the start time denotes the point at which subjects entered the cohort. The end time is either March 31, 2017 or July 31, 2019, with more details provided in Section 4.1. The end event refers to the occurrence of the target disease. The parameters of the Cox model are defined as follows.

**Fig 2:**
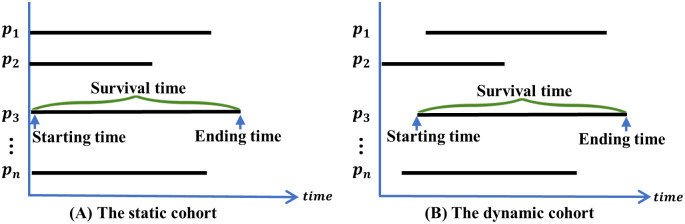
Examples of the static and dynamic cohort survival times. The static cohorts have a unified starting time; Subjects in dynamic cohorts with sequential enrolling time. For the notation of $p_{i_{|i\in R^{+}}}$ denotes the *i*-*th* specific subject.

*D*efinition 1 *Survival time*. Survival time defines the period between the starting and ending points. The starting time varies by the cohort study type. In static cohorts, the starting time is the same for all participants and is defined as the beginning of the study. In contrast, in dynamic cohorts, each subject enrolls at a different time, which is defined as the starting time. The ending time represents the occurrence of the event of interest, such as the onset, recurrence, or death. In this study, data from both the UK Biobank’s dynamic cohort and the Weihai physical examination dynamic cohort are utilized. Therefore, survival time is defined as the time from the cohort’s sequential enrollment to the event of interest. To clarify the definition of survival time, an example is illustrated in the Fig 2.

**Fig 3:**
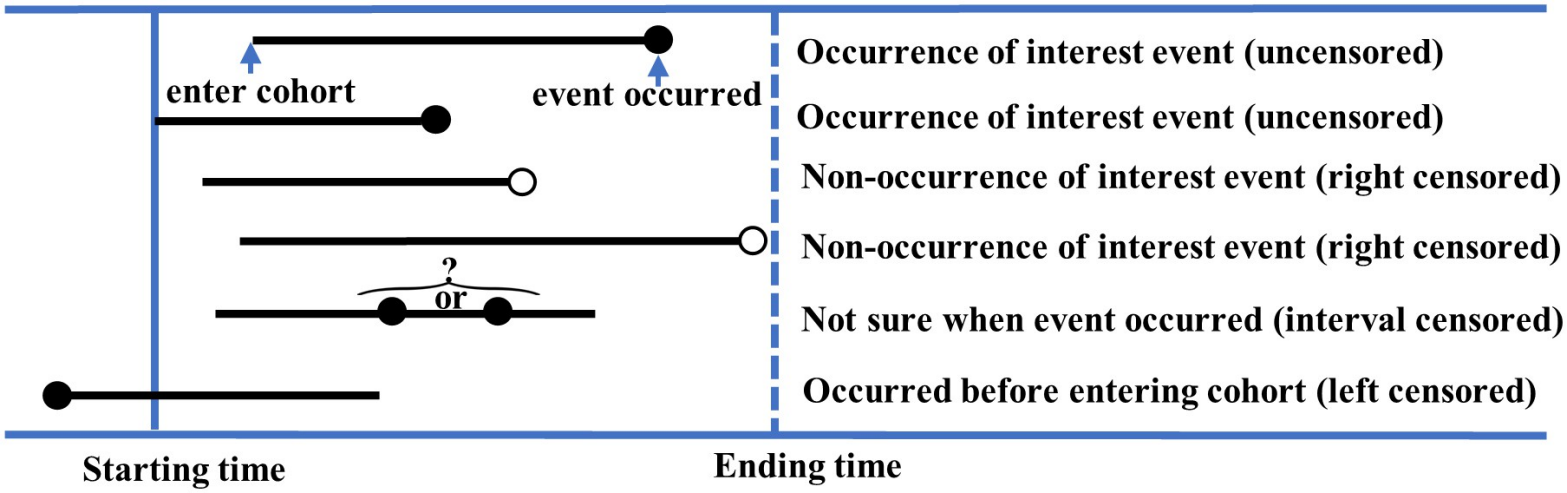
An illustration demonstrating the censoring definition.

*D*efinition 2 *Censored data*. Subjects are considered censored when information about the event is not available at the time of occurrence due to loss to follow-up or non-occurrence of the outcome event before the trial ends [43]. Censored data can be categorized into three groups: left-censored, right-censored, and interval-censored. The left-censored group includes subjects who were at risk of developing the disease before entering the cohort. Since the Cox model can only handle right-censored data, these left-censored subjects are excluded at the beginning of the study. The right-censored group includes subjects who did not experience the event of interest by the endpoint of the trial. This study did not involve interval-censored data. An example is shown in Fig 3 to clarify the definition of censoring.

*D*efinition 3 *Uncensored data*. Uncensored data refer to events of interest that occurred during the trial period, and we know the specific point in time. In this study, uncensored data represent new cases during the trial period. We introduced a censoring indicator *δ*_*i*_ ∈ {0, 1} to indicate the censored type of data (i.e., *δ*_*i*_ = 1 stands for an uncensored instance, and *δ*_*i*_ = 0 means a censored instance).

*D*efinition 4 *Features*. Features represent attributes, properties, or characteristics of objectives [44].

*D*efinition 5 *Survival and hazard function*. The survival function *S* (*t*) represents the probability that a subject’s survival time that *T* is not earlier than a specific time *t*. Mathematically, the survival function can be expressed as follows:

$$\begin{array}{c} S\;(t)=P\;(T>t). \end{array}$$ (1)

Another commonly used function in survival analysis is the hazard function *h*(*t*), which can be interpreted as the probability that a subject survives at time *t* and dies in *t*+Δ*t* (*t* > 0). The hazard function is given by

$$\begin{array}{c} h(t)=\underset{\Delta t\to 0}{\text{lim}}\;\frac{P(t\leq T<t+\Delta t|T\geq t)}{\Delta t}. \end{array}$$ (2)

*D*efinition 6 *Baseline*. The baseline is denoted as the beginning time of conducting the related research [45].

#### Multitask learning

*D*efinition 7 *Multitask Learning*. Given a set of *K* learning tasks, either all or a subset of which may be related, the goal of multitask learning is to train a model to learn all *K* tasks simultaneously, while utilizing the knowledge and information present in other tasks, to enhance the learning of each task’s model [28].

Multitask learning, a form of inductive transfer proposed by Caruana [27], is a powerful branch of machine learning that involves jointly training multiple tasks simultaneously, to utilize the correlation among different tasks. This approach can benefit the learning of model parameters by leveraging the knowledge contained across all tasks, resulting in improved learning efficiency and acting as a regularizer to prevent overfitting [28].

#### Disease risk metrics for personalized prediction

The individual risks are measured by the following metrics:

*D*efinition 8 *Basis risk* is the incidence rate of each age group within a certain period of time (e.g., 5 years, 10 years) representing the average risk level of disease in the same population and forms an important basis for classifying the risk level.

*D*efinition 9 *Relative risk (RR)* is an indicator of the strength of the association between exposure and morbidity (mortality), which cannot be used as a predictor of disease risk.

*D*efinition 10 *Absolute risk (AR)* denotes the probability that an individual with specific risk factors does not have the studied outcome (e.g., stroke) at age *a*, but the outcome occurs at age (a+T), where T is the artificially defined follow-up time.

*D*efinition 11 *Relative absolute risk (RAR)* is the ratio of the absolute risk of an individual to the average absolute risk of the same age group for given risk factors, reflecting that the absolute risk of each individual is a multiple of the average absolute risk of the population of the same age group.

*D*efinition 12 *Excess absolute risk (EAR)* is the difference between the absolute risk of an individual and the average absolute risk (i.e., the population average basis risk) of the same age group for given risk factors, reflecting the difference between the absolute risk of each individual and the average absolute risk of the same age group.

#### *L*_2,1_ norm-based regularizer

Overfitting is a common problem for model training [46], which can be diluted by utilizing the *L*_2,1_ norm regularizer to alleviate the negative impact. The *L*_2,1_ norm *de facto* was proposed based on the *L*_1_ norm and *L*_2_ norm. In machine learning, *L*_1_ norm enforces sparsity in models, and *L*_2_ norm penalties in some sense discourage sparsity. For a greater level of understanding of *L*_2,1_ norm-based regularizer, we introduce the *L*_1_ norm and *L*_2_ norm at the beginning of the following section.

*L*_1_ **norm**. The *L*_1_ norm is defined as the sum of the absolute values of the elements of the parameter vector. The mathematical formula is as follows:

$$\begin{array}{c} {\left\|x\right\|}_{1}=\sum_{i=1}^{I}|x_{i}|, \end{array}$$ (3)

where ***x*** denotes the parameter vector, *x*_*i*_ represents the *i*-*th* element of the parameter vector, and *I* is the total number of elements of vector ***x***.

*L*_2_ **norm**. The *L*_2_ norm is the square root of the inner product of the vector. The mathematical formula is given as follows:

$$\begin{array}{c} {\left\|x\right\|}_{2}=\sqrt{\sum_{i=1}^{I}x_{i}^{2}.} \end{array}$$ (4)

*Definition* 13 *L*_2,1_ *norm*. The *L*_2,1_ norm is the sum of the *L*_2_ norm of all rows in matrix ***X***, defined by

$$\begin{array}{c} {\left\|X\right\|}_{2,1}=\sum_{p=1}^{P}{\left\|x^{p}\right\|}_{2}, \end{array}$$ (5)

where *p* is the *p*-*th* row of ***X***.

The *L*_2,1_ norm first computes the *L*_2_ norm of the matrix ***X*** row vector and then computes the *L*_1_ norm of all *L*_2_ norm. The *L*_2_ norm promotes a dense (non-zero) solution within each row, and the outer *L*_1_ norm forces the *L*_2_ norm of some rows to be zero, which means that all values in that row are zero. The *L*_2,1_ norm regularization, therefore, reduces the weights of some rows to zero, which serves the purpose of feature selection [47].

## The model

Here, we introduce our model framework in detail. We employed the model architecture from a unified multitask survival analysis framework to develop MTL-Cox [48]. MTL-Cox consists of two parts: 1) The Multiple Cox model is used for multiple chronic diseases and 2) regularization terms make multiple diseases share parameters.

### Cox model

The Cox model is expressed by the hazard function *h*(*t*). The Cox model can be represented with the feature matrix ***X*** = {***x***_1_, ***x***_2_, ***x***_3_, …, ***x***_*P*_} as

$$\begin{array}{c} h(t;X)=h_{0}(t)exp(\beta_{1}x_{1}+\cdots +\beta_{P}x_{P}). \end{array}$$ (6)

In the above equation, *h*_0_(*t*) represents the baseline hazard function when the feature vector is 0. The exponential function is denoted by *exp* (*exp*(*x*) = *e*^*x*^). The coefficients ***β*** = {*β*_1_, *β*_2_, …, *β*_*P*_} measure the impact, or effect size, of each feature. *h*_0_(*t*) does not need to follow a specific distribution, and it cannot be estimated [49]. Therefore, *h*_0_(*t*) is considered nonparametric. However, the exponential part (i.e., *exp*(*β*_1_***x***_1_ + ⋯ + *β*_*P*_***x***_*P*_)) takes the form of a parametric model, where the parameters can be estimated from the observed feature values of the subjects [49].

Parameter estimates in the Cox model are obtained by maximizing the partial likelihood. First, let *T*_1_ ≼ *T*_2_ ≼ ⋯ ≼ *T*_*N*_ be the ordered time to the event of interest for *N* subjects. For each survival time *T*_*i*_, all subjects with a survival time greater than *T*_*i*_ constitute a risk set, denoted as *R*(*T*_*i*_). Subjects in *R*(*T*_*i*_) survived before *T*_*i*_, but were still at risk of experiencing the event of interest. Thus, the partial likelihood function is formalized in Eq (7):

$$\begin{array}{c} l\left(\beta \right)=\prod_{i=1}^{N}{\left[\frac{exp(\sum_{p=1}^{P}x_{ip}\beta_{p})}{\sum_{s\in R(T_{i})}exp(\sum_{p=1}^{P}x_{sp}\beta_{p})}\right]}^{\delta_{i}}, \end{array}$$ (7)

where *δ*_*i*_ = 1 represents that a subject falls ill at time *T*_*i*_, *δ*_*i*_ = 0 represents a censored subject, *P* is the number of features, and *s* denotes subjects in the risk set *R*(*T*_*i*_). Finally, the coefficients can be estimated by minimizing the negative log partial likelihood:

$$\begin{array}{c} \text{ln}\;l(\beta )=-\sum_{i=1}^{N}\delta_{i}[(\sum_{p=1}^{P}x_{ip}\beta_{p})-ln\sum_{s\in R(T_{i})}exp(\sum_{p=1}^{P}x_{sp}\beta_{p})]. \end{array}$$ (8)

The first derivative function is

$$\begin{array}{c} {\text{ln}}^{\prime }\;l(\beta )=-\sum_{i:{\;\delta }_{i}=1\;}{(X}_{i}-\frac{\sum_{s\in R(T_{i})}exp(X_{s}\beta )X_{s}}{\sum_{s\in R(T_{i})}exp(X_{s}\beta )}), \end{array}$$ (9)

and the Hessian matrix of the negative log partial likelihood is

$$\begin{array}{cc} {\text{ln}}^{\prime \prime }\;l(\beta ) & =-\sum_{i:{\;\delta }_{i}=1\;}(\frac{\sum_{s\in R(T_{i})}exp(X_{s}\beta )X_{s}X_{s}^{T}}{\sum_{s\in R(T_{i})}exp(X_{s}\beta )}-\\ & \frac{\left[\sum_{s\in R(T_{i})}exp(X_{s}\beta )X_{s}][\sum_{s\in R(T_{i})}exp(X_{s}\beta )X_{s}^{T}\right]}{{\left[\sum_{s\in R(T_{i})}expX_{s}\beta )\right]}^{2}}), \end{array}$$ (10)

where $X_{s}\in R^{1\times P}$ denotes the feature values of the *s*-*th* subject, and $X_{s}^{T}$ is the transpose of ***X***_*s*_. Using the first derivative function and Hessian matrix, we can minimize the negative log partial likelihood.

The traditional Cox model is a well-recognized statistical technique for exploring the relationship between the survival of a subject and several features. However, the traditional Cox model belongs to the category of single-task learning and ignores the relationship between multiple diseases. To solve the above issues, we propose the multitask Cox model, which learns the survival analysis problems of multiple related chronic diseases in parallel.

### Multitask Cox model

#### Framework

The chronic diseases studied in this research share common risk factors, and therefore, we used parameter-based multitask learning with a low-rank assumption to estimate the coefficients of different chronic disease predictors that share a low-dimensional subspace [28].

**Fig 4:**
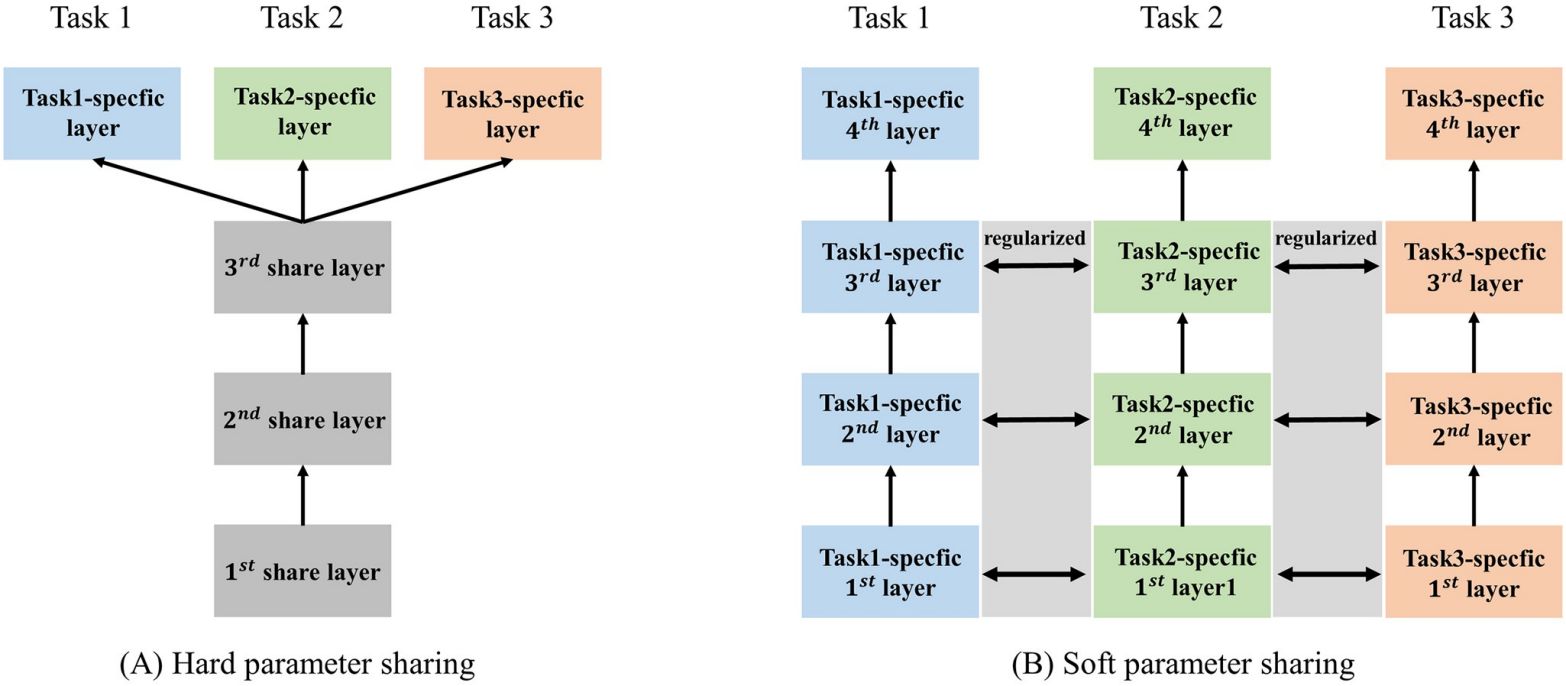
Strategies of parameter sharing in the multitask learning framework. (A) Hard parameter-sharing mechanism transfers the parameter training representations from hidden layers across all tasks. (B) The soft parameter sharing mechanism shares penalized information between tasks by regularization.

Parameter-based multitask learning can be classified into two sub-categories: hard parameter-sharing and soft parameter-sharing mechanisms. The hard parameter-sharing mechanism shares the hidden layers between all tasks while keeping several task-specific output layers [50], as shown in Fig 4. However, since all tasks need to use the same set of parameters on shared-bottom layers, the hard parameter-sharing mechanism has optimization conflicts caused by task differences [51]. On the other hand, the soft parameter-sharing mechanism is associated with the identity parameters in each task. The differences between the parameters of the model are then regularized to encourage sharing of knowledge among the parameters [50]. For example, Obozinski et al. [52] used the *L*_2,1_ norm for regularization, and the trace norm is also a common regularized term suitable for MTL [53]. The soft parameter-sharing mechanism is regularized by sharing prior parameters to train stabilities while forgetting overly strong task-related constraints. Therefore, we developed the multitask Cox model using the soft parameter-sharing mechanism as the learning strategy.

A general regularized empirical loss paradigm for MTL can be formulated as

$$\begin{array}{c} \text{arg}\;\underset{B}{\text{min}}\;L(B)+R(B). \end{array}$$ (11)

In this paper, a chronic disease is a task. Cox models of multiple chronic diseases are denoted by *L*(***B***); that is, $L\left(B\right)=\;\sum_{k=1}^{K}\frac{\text{ln}\;l(\beta_{k})}{N_{k}}$. ***β***_*k*_, *N*_*k*_, and ln *l*(***β***_*k*_) represent the parameters to be estimated, the number of training instances, and the empirical loss on the training set with respect to the *k*-*th* task, respectively. $B=\;\left[\beta_{1},\beta_{2},\;...,\;\beta_{K}\right]R^{P\times K}$, the *k*-*th* column of ***B*** (i.e., $\beta_{k}R^{P\times 1}$) denotes the parameters to be estimated for the *k*-*th* task. *R*(***B***) is the regularization term to encode the task relatedness.

The *L*_2,1_ norm combines the strengths of the *L*_1_ and *L*_2_ norms, allowing the model to generalize in both sparse and dense spaces. And the *L*_2,1_ norm can be expressed using the Eq 12:

$$\begin{array}{c} R(B)={\left\|B\right\|}_{2,1}=\sum_{p=1}^{P}{\left\|\beta^{p}\right\|}_{2}=\sum_{p=1}^{P}\sqrt{\sum_{k=1}^{K}\beta_{p,k}^{2}}\;, \end{array}$$ (12)

where ***β***^*p*^, the *p*-*th* feature of all tasks, is the *p*-*th* row of ***B***.

When the number of tasks is one, matrix ***B*** only has a single column. In this scenario, Eq (12) can be expressed as the *L*_1_ norm regularization optimization problem, where it equals the sum of absolute values in vector ***β***. On the other hand, if there are multiple tasks, the *L*_2,1_ norm of ***β***^*p*^, combines the parameters for the *p*-*th* feature of all tasks. This type of regularization takes into account the relationship between multiple tasks to select features instead of solely relying on the strength of a single input variable, as single-task learning does. Therefore, the *L*_2,1_ norm regularization can learn multiple tasks simultaneously and synthesize the information from various tasks to enhance the model’s performance.

To maintain a balance between *L*(***B***) and *R*(***B***), a positive regularization parameter, λ, is introduced. Consequently, the loss function of the proposed multitask Cox model can be expressed as:

$$\begin{array}{c} \underset{B}{\text{min}}\;\sum_{k=1}^{K}\frac{\text{ln}\;l(\beta_{k})}{N_{k}}+\lambda \sum_{p=1}^{P}\sqrt{\sum_{k=1}^{K}\beta_{p,k}^{2}}\;, \end{array}$$ (13)

where ln *l*(***β***_*k*_) is the loss function of the Cox model for the *k*-*th* task; that is,

$$\begin{array}{c} \text{ln}\;l(\beta_{k})=-\sum_{i=1}^{N}\delta_{i,k}[(\sum_{p=1}^{P}x_{ip,k}\beta_{p,k})-\text{ln}\;\sum_{s\in R(T_{i})}exp(\sum_{p=1}^{P}x_{sp,k}\beta_{p,k})]. \end{array}$$ (14)

#### Optimization

In this section, we present the details of learning and optimizing the model.

As the Hessian matrix of the negative log partial likelihood is nonnegative and the *L*_2,1_ regularization norm is convex [54], the objective function Eq (11) is guaranteed to be convexity. Furthermore, since the *L*_2,1_ norm is non-smooth [55], the proximal gradient has been employed to optimize the model along with converging at a fast rate O(1/ε) [56].

Choosing the initial ***B***^(0)^ and repeating for *j* = 1, 2, 3, …

$$\begin{array}{c} B^{\left(j\right)}={prox}_{t_{j}}(B^{\left(j-1\right)}-t_{j}\nabla L(B^{\left(j-1\right)})), \end{array}$$ (15)

where

$$\begin{array}{c} {\text{prox}}_{t}=\text{arg}\;\underset{B}{\text{min}}\;\frac{1}{2}{\left\|B-G\right\|}_{2}^{2}+\lambda {\left\|B\right\|}_{2,1}, \end{array}$$ (16)

where ***G*** is gradient step; that is, $G\;=S-\;\frac{1}{\gamma }\nabla L(S$). In addition, *γ* is the step size, and ***S*** is the current search point. ***S*** can be written as

$$\begin{array}{c} S^{\left(j\right)}=\;B^{\left(j\right)}+t_{j}\left({B^{\left(j\right)}-B}^{\left(j-1\right)}\right), \end{array}$$ (17)

*t*_*j*_ denotes the combination scalar of previous points. ∇*L*(***S***) is the gradient of the loss function at search point ***S***. For all *K* tasks, ∇*L*(***S***) is formulated as

$$\begin{array}{c} \nabla L(S)=[\frac{{\text{ln}}^{\prime }\;l(S_{1})}{N_{1}},\frac{{\text{ln}}^{\prime }\;l(S_{2})}{N_{2}},\ldots ,\frac{{\text{ln}}^{\prime }\;l(S_{K})}{N_{K}}], \end{array}$$ (18)

***S***_*i*_ is the parameter to be estimated for the *i*-*th* task, and is given in the *i*-*th* column of ***S***. *N*_*i*_ denotes the number of training instances of the *i*-*th* task. ln′ *l*(***S***_*i*_) is the derivative of the negative log partial likelihood, and all tasks ln′ *l*(***S***_*i*_) share the same formulation:

$$\begin{array}{c} {\text{ln}}^{\prime }\;l(\beta )=-\sum_{i=1}^{N}\delta_{i}[\sum_{p=1}^{P}x_{ip}-\frac{\sum_{s\in R(T_{i})}X_{s}exp(X_{s}\beta )}{\sum_{s\in R(T_{i})}exp(X_{s}\beta )}]. \end{array}$$ (19)

In citing from [57], we derived the following theorem refereed the convergence of proximal gradient descent:

**Theorem 1** Let $f\left(B\right)=\underset{B}{\text{min}}\;L(B)+\lambda R(B)$. Proximal gradient descent with the fixed step size *t* ≤ 1/*L* satisfies the following Eq (20)

$$\begin{array}{c} f(B^{\left(j\right)})-f*\leq \frac{{\|B^{\left(0\right)}-B*\|}_{2}^{2}}{2tj}. \end{array}$$ (20)

The proximal gradient descent converge in the rate of O(1/j) inference from Eq (20). The detailed proof procedure is available in [57]. The pseudo-code of the MTL-Cox model is presented in Algorithm 1.

**Algorithm 1**: Proximal gradient algorithm used for model learning.

**Input**: Feature matrix ***X***, initial coefficient matrix ***B***^(0)^, and corresponding regularization scales.

**Output**: $\hat{B}$

1: **Initialization**: ***B***^(1)^ = ***B***^(0)^, *q*_−1_ = 0, *q*_0_ = 1, *γ*_0_ = 1,and *j* = 1;

2: **repeat**

3:  Set $t_{j}=\frac{q_{i-2}-1}{q_{i-1}},S^{\left(j\right)}=\;B^{\left(j\right)}+t_{j}\left({B^{\left(j\right)}-B}^{\left(j-1\right)}\right)$;   // the current search point is defined as a combination of the previous two search points

4:  **for** *i* = 1, 2, … **do**

5:   Set *γ* = 2^*i*^*γ*_*j*−1_;   // select the optimal step size by the line search strategy

6:   $B^{\left(j\right)}={prox}_{t_{j}}(B^{\left(j-1\right)}-t_{j}\nabla L(B^{\left(j-1\right)}))$;   // Proximal gradient descent

7:   **if** $\left(B^{\left(j+1\right)})\leq \prod_{\gamma ,S^{\left(i\right)}}(B^{\left(i+1\right)}\right)$ **then**

8:    *γ*_*j*_ = *γ*, **break**

9:   **end if**

10:  **end for**

11:  $q_{j}=\frac{1+\sqrt{1+4q_{j-1}^{2}}}{2}$;

12:  *j*++;

13: **until** Convergence of ***B***^(*j*)^;

14: $\hat{B}=B^{\left(j\right)}$

## Materials

In this paper, we first constructed an MTL-Cox model for nine chronic diseases based on the UK Biobank dataset. Subsequently, we built another MTL-Cox model for five cancers within these chronic diseases using the Weihai physical examination dataset to validate the effectiveness of the MTL-Cox model framework. Here, we first describe the dataset used in our experiment and demonstrate the process of feature selection based on the UK Biobank dataset. More details about the Weihai physical examination dataset can be found in the Supplementary file S1 File.

### Ethics statement

The study protocol was approved by the North West Multi-center Research Ethics Committee, the Patient Information Advisory Group in England and Wales, and the Community Health Index Advisory Group in Scotland. All participants in the surveys gave their informed consent. The patients/participants provided their written informed consent to participate in this study.

### Datasets

The data utilized in this study were obtained from the UK Biobank, a dataset for cohort studies that was used to evaluate the MTL-Cox model’s ability to predict chronic diseases. The UK Biobank recruited 500,000 participants between 2006 and 2010, ranging in age from 37 to 73 years old [58–60]. Participants attended their closest of 22 assessment centers across England, Wales, and Scotland during a baseline assessment visit, which included information on their socioeconomic and demographic characteristics, general health and medical history, lifestyle, and diet. All participants provided written informed consent, and the study protocol was approved by the North West Multi-Centre Research Ethics Committee.

**Fig 5:**
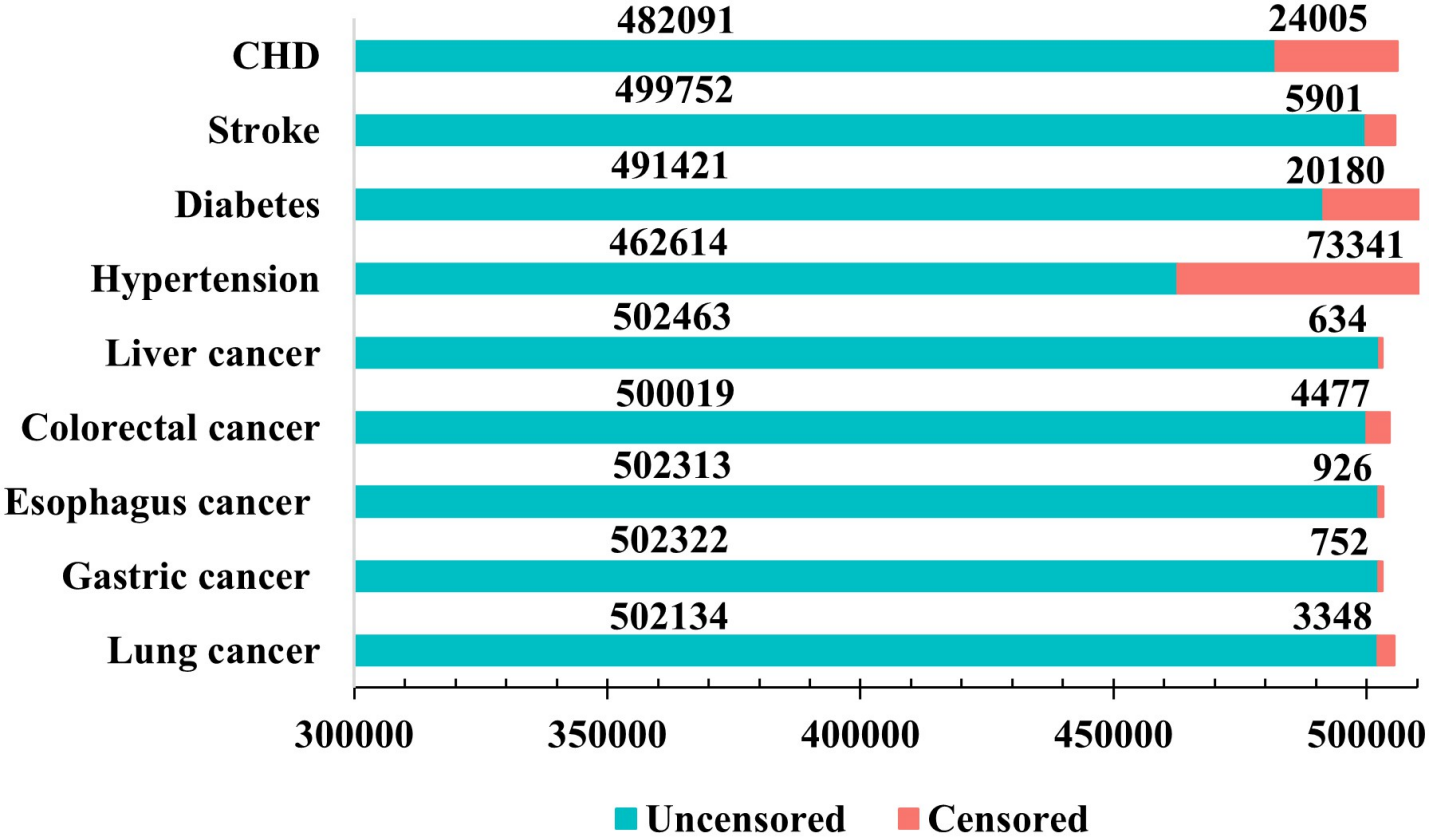
The censored statistics of the nine chronic diseases. The definitions of censored and uncensored are presented in the section Preliminaries.

Following the 2020 World Health Organization report on chronic diseases [61], we selected nine typical chronic diseases for our study, including lung cancer, gastric cancer, esophageal cancer, colorectal cancer, liver cancer, hypertension, diabetes, stroke, and *c*oronary *h*eart *d*isease (CHD). The cancer records for the UK Biobank cohort were mostly completed by March 2017 (https://epi.grants.cancer.gov/cohort-consortium/members/ukb.html. Accessed on Mar. 29^*th*^, 2022; https://www.nature.com/collections/bpthhnywqk. Accessed on Mar. 29^*th*^, 2022.). Therefore, to improve prediction accuracy, we defined the follow-up time as follows: participant follow-up began at the time of inclusion in the UK Biobank study. We categorized the end date of follow-up into two cases: for cancers, participant records were available until either March 31, 2017, or until the patient was diagnosed with cancer. However, for other diseases (i.e., hypertension, diabetes, stroke, and CHD), participant follow-up ended on July 31, 2019. We excluded participants who had been diagnosed with the target disease before entering the cohort. Follow-up was censored if events of interest were not observed for various reasons. The censored statistics of the nine chronic diseases are shown in Fig 5. We defined the nine chronic disease codes using the International Classification of Diseases, 10th edition (ICD-10), as follows: lung cancer, C34; gastric cancer, C16; esophageal cancer, C15; colorectal cancer, C18-C20; liver cancer, C22; hypertension, I10-I15; diabetes, E10-E14; stroke, I60-I64; and CHD, I20-I25.

### Feature selection

Based on expert knowledge, we acquired 78 features from the UK Biobank dataset, comprising 58 clinical features and 20 demographic features. However, the original encoding of categorical variables was not suitable for data analysis, thus we recoded them (as listed in S15 Table). Additionally, the continuous variables were standardized. Detailed information on demographic and clinical features can be found in the Supplementary file S1 Table, while baseline information for the nine chronic diseases is presented in the Supplementary file S2 Table.

**Fig 6:**
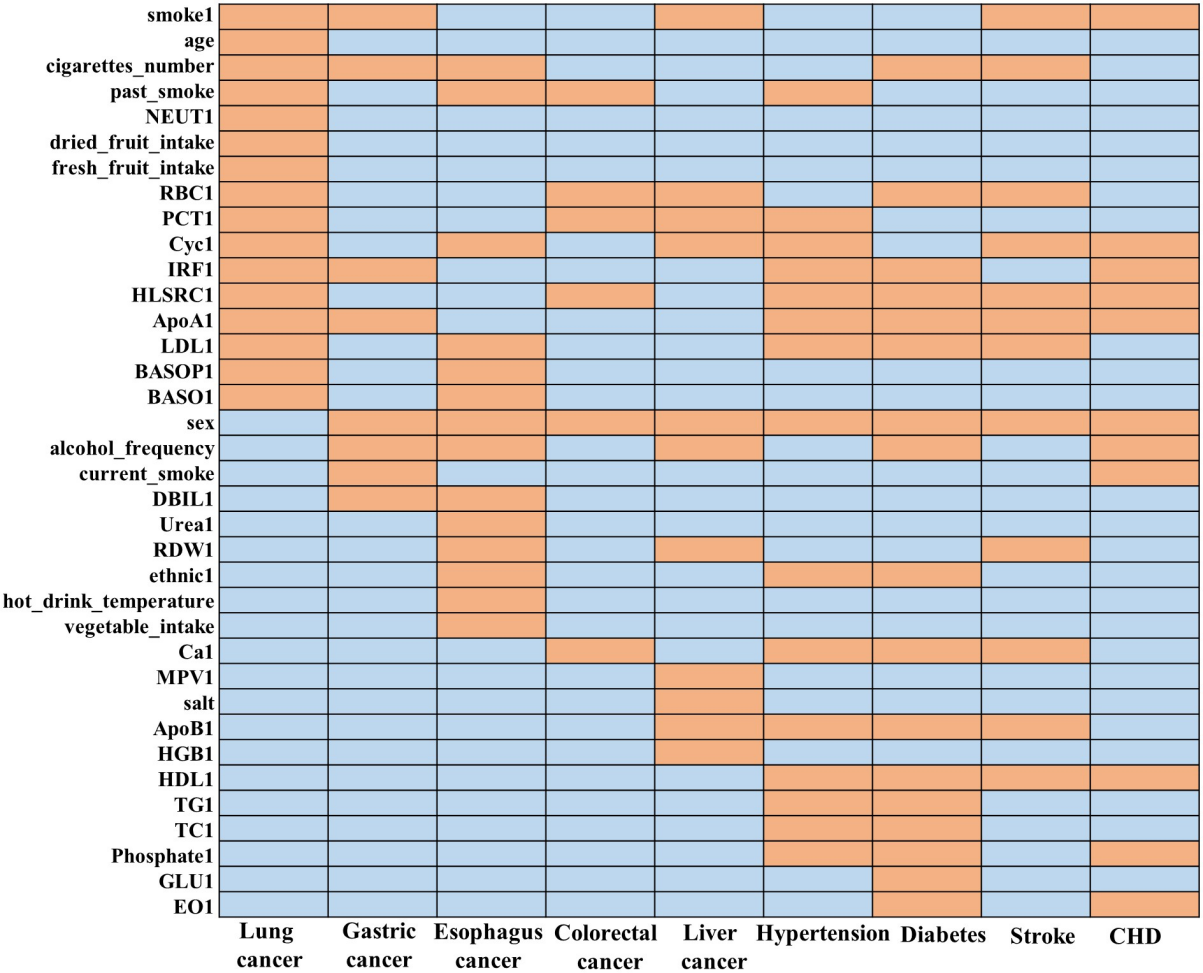
The selected features of nine chronic diseases. The color of orange represents the selected features of the corresponding disease; other features are represented in the color of blue.

It is important to note that when it comes to disease prediction models, having more features does not necessarily mean better performance. We prefer using a limited number of features that produce the best prediction outcomes. This approach not only reduces the cost and effort required to collect subject information but also reduces computational requirements and time. Hence, we used mono-factor analysis and forward regression sequentially to select 36 features related to chronic diseases from the 78 features (as shown in Fig 6). Furthermore, the summary of selected features is given in Table 2. The union of the nine disease variables was used as input features for MTL-Cox.

**Table 2:** A summary description of 36 chronic diseases related selected features.

Name	Description	Name	Description
smoke1	Smoking status	sex	Gender
age	Age at recruitment	alcohol_frequency	Alcohol intake frequency
cigarettes_number	Number of cigarettes currently smoked daily	current_smoke	Current smoking status
past_smoke	Past tobacco smoking	DBIL1	Direct bilirubin
NEUT1	Neutrophill count	Urea1	Urea
dried_fruit_intake	Dried fruit intake	RDW1	Red blood cell distribution width
fresh_fruit_intake	Fresh fruit intake	ethnic1	Ethnic background
RBC1	Red blood cell (erythrocyte) count	hot_drink_temperature	Hot drink temperature
PCT1	Platelet crit	vegetable_intake	Vegetable intake
Cyc1	Cystatin C	Ca1	Calcium
IRF1	Immature reticulocyte fraction	MPV1	Mean platelet (thrombocyte) volume
HLSRC1	High light scatter reticulocyte count	salt	Salt added to food
ApoA1	Apolipoprotein A	ApoB1	Apolipoprotein B
LDL1	LDL direct	HGB1	Hemoglobin concentration
BASOP1	Basophill percentage	HDL1	HDL cholesterol
BASO1	Basophill count	TG1	Triglycerides
TC1	Cholesterol	Phosphate1	Phosphate
GLU1	Glucose	EO1	Eosinophill count

## Experiments and results

Here, we introduce the main performance metrics in the survival analysis and compare the performance with other survival analysis models.

### Performance metrics

Because of the existence of right-censored data in the survival data, the standard evaluation metrics used for regression analysis, such as a sum of the mean square errors *R*^2^ and root mean squared error, are not used for measuring the prediction performance of survival analysis models. Therefore, we used five metrics (i.e., concordance, AUC, specificity, sensitivity, and Youden index) to comprehensively evaluate the performance of the model. These metrics are commonly used in survival analysis.

#### Concordance index

**Fig 7:**
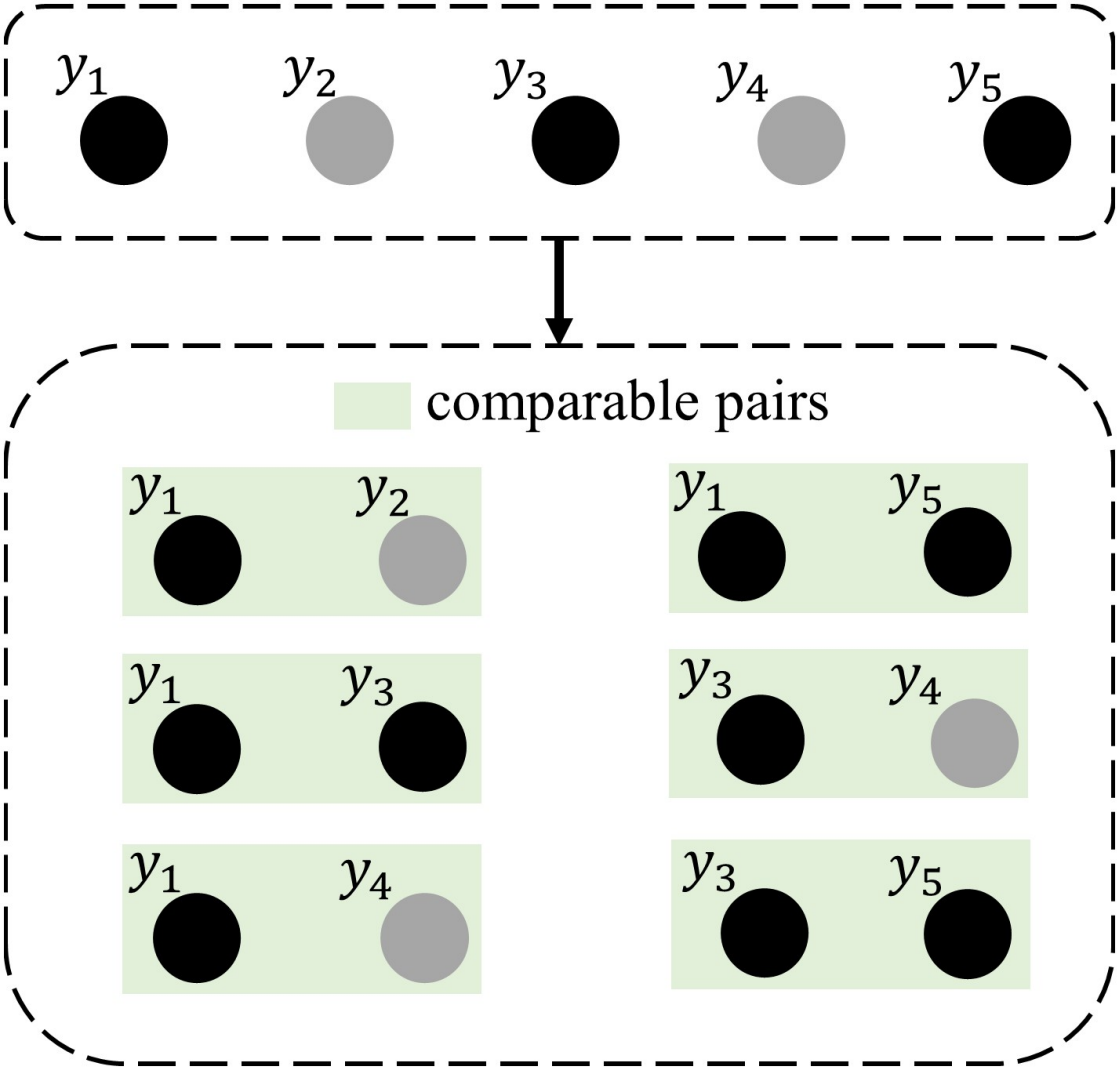
An example of calculating the number of comparable pairs for the C-index (*y*_1_ ≼ *y*_2_ ≼ *y*_3_ ≼ *y*_4_ ≼ *y*_5_). ≼ based on the timeline. Black circles denote uncensored observations, and gray circles indicate censored observations.

The concordance index (C-index), proposed by Franke Harrell Jr., is an evaluation metric commonly used in survival analysis [62, 63]. It measures the proportion of pairs of patients for whom the predicted outcomes are concordant with the actual outcomes. In other words, it estimates the probability that the predicted outcome will match the actual observed outcome. The C-index can be mathematically expressed as follows:

$$\begin{array}{c} C=P({\hat{y}}_{1}>{\hat{y}}_{2}|y_{1}>y_{2}), \end{array}$$ (21)

where ${\hat{y}}_{i}\left(i=1,2\right)$ is the predicted value, and *y*_*i*_(*i* = 1, 2) is the actual observation value. In the survival analysis model, subjects with a lower risk should survive longer, so the C-index can be computed using

$$\begin{array}{c} \hat{C}=\frac{1}{n}\sum_{i:\delta_{i}=1}\sum_{j:y_{i<}y_{j}}I(x_{i}\hat{\beta }>x_{j}\hat{\beta }), \end{array}$$ (22)

where *n* represents the number of comparable pairs, *I* denotes the indicator function, and $\hat{\beta }$ represents the vector of estimated parameters. The calculation example of *n* is illustrated in detail in Fig 7. The C-index ranges from 0.5 to 1, where a C-index of 0.5 represents random prediction and implies that the model has no predictive effect. On the other hand, a C-index of 1 indicates perfect consistency, suggesting that the model’s predictions match the actual outcomes completely.

#### AUC

*A*rea *U*nder the *C*urve (AUC) is a commonly used evaluation metric for measuring the performance of binary classification models. It is calculated as the area under the *R*eceiver *O*perating *C*haracteristic (ROC) curve. The ROC curve plots the true positive rate (sensitivity) on the y-axis and the false positive rate (1-specificity) on the x-axis. AUC is preferred as an evaluation criterion for models because sometimes the ROC curve does not clearly indicate which classifier performs better. AUC can be calculated by determining the area under the ROC curve. The value range for AUC is between 0.5 and 1, where a higher AUC indicates better model performance.

#### Specificity, sensitivity, and Youden index

The performance of models can also be evaluated using sensitivity, specificity, and the Youden index:

- Specificity: refers to the fraction of those without the disease who will have a negative test result. According to the definition of specificity, the value of specificity is expressed as
  $$\begin{array}{c} Specificity=\frac{TN}{FP+TN}, \end{array}$$ (23)
  where *TN* denotes the number of true-negative cases, *FP* is the number of false-positive cases. In addition, 1-*Specificity* represents the misdiagnosis rate.
- Sensitivity: refers to the proportion of patients who are actually sick that the model can correctly determine as patients. The sensitivity can be calculated via the following formula:
  $$\begin{array}{c} Sensitivity=\frac{TP}{TP+FN}, \end{array}$$ (24)
  where *TP* is truly positive, and *FN* denotes false negative. Missed diagnosis rate can be formulated as 1-*Sensitivity*.
- Youden index: represents the model’s total ability to discover real patients and non-patients. An excellent model should keep both missed and misdiagnosed rates as low as possible, and the Youden index takes these two aspects into account. The Youden index can be computed as
  $$\begin{array}{c} Youden=Specificity+Sensitivity-1. \end{array}$$ (25)

The higher the Youden index, the better the performance of the model, and the greater the degree of authenticity.

### Competing methods

The performance of the MTL-Cox model was compared to several single-task learning survival analysis models, including Cox, the Weibull regression model, and Cox-LASSO. This section provides the details of the Weibull regression and Cox-LASSO models.

A. Weibull regression model

The Weibull regression model is a type of parametric survival analysis model [64]. In this model, the hazard function in proportional hazards can be expressed by [65]

$$\begin{array}{cc} h(t,x,\beta ,\lambda ) & =\lambda t^{\lambda -1}e^{-\lambda (\beta_{0}+x\beta )}\\ & =\lambda t^{\lambda -1}e^{-\lambda \beta_{0}}e^{-\lambda \beta^{\prime }x^{\prime }}\\ & =\lambda \gamma t^{\lambda -1}e^{-\lambda \beta^{\prime }x^{\prime }}\\ & =h_{0}(t)e^{\theta x^{\prime }}, \end{array}$$ (26)

where ***x*** is a vector with dimensions 1 × *p*, ***β*** is a vector with dimensions *p* × 1, and *p* represents the *p*-*th* feature. The baseline hazard function is *h*_0_(*t*) = λ*γt*^λ−1^, while the scale parameter is represented by $\gamma =\frac{1}{\sigma }$ and the shape parameter by λ. The parameter ***θ***_1×*p*_ has a hazard ratio interpretation for subjects. The ***β*** vector is estimated by maximum likelihood estimation.

B. Cox-LASSO

Cox-LASSO regression can refine the model by constructing a penalty function [66, 67]. Specifically, the LASSO technique minimizes the residual sum of squares while imposing a constraint on the sum of the absolute coefficients. This approach helps to address the issue of multicollinearity and leads to the better predictive performance of the model [66]. The expressions for the Cox-LASSO and Cox models are the same, as shown in Eq (6), but the estimated coefficients, ***β***, differ between the two models. In the Cox model, maximum likelihood estimation is used to estimate the coefficients ***β***. On the other hand, in the Cox-LASSO model, ***β*** is estimated by introducing lasso term constraints proposed by Tibshirani in 1997 [66], as shown in Eq (27).

$$\begin{array}{c} \hat{\beta }\;=arg\underset{B}{\text{min}}\;\text{ln}\;l(\beta ),\text{subject}\;\text{to}\sum |\beta_{p}|\leq s, \end{array}$$ (27)

where *s* is a specified parameter following the constraint of *s* > 0 [66], *l* (***β***) stands for the log partial likelihood (Eq (7)), and ln *l* (***β***) denotes the partial likelihood function that is denoted as:

$$\begin{array}{c} \text{ln}\;l(\beta )=\sum_{i=1}^{N}\delta_{i}[(\sum_{p=1}^{P}x_{ip}\beta_{p})-\text{ln}\;\sum_{s\in R(T_{i})}exp(\sum_{p=1}^{P}x_{sp}\beta_{p})]. \end{array}$$ (28)

We divided the complete dataset into five non-intersecting subsets and applied the 5-fold cross-validation technique to train both MTL-Cox and competing methods. Specifically, we used one subset for validation and the other four subsets for training. To assess the performance of MTL-Cox against other single-task learning approaches, we conducted a paired-sample Wilcoxon signed-rank test to quantitatively evaluate the concordance index, AUC, specificity, sensitivity, and Youden index.

The MTL-Cox model has three hyper-parameters: the number of searching parameters, the rate of the smallest search parameter compared to the largest one, and the scale of the first searching point; these were set as 50, 0.01, and 1, respectively. We used the personalized disease evaluation system proposed in this paper using the MTL-Cox model to achieve personalized disease risk prediction. In our experiment, we implemented the MTL-Cox model via Matlab and the personalized prediction in R (The code can be accessed through the URL https://github.com/Shuaijiea/paper-code.).

### Results

The results obtained from the UK Biobank dataset are presented below, while those obtained from the Weihai physical examination dataset can be found in Supplementary file S1 File.

**Fig 8:**
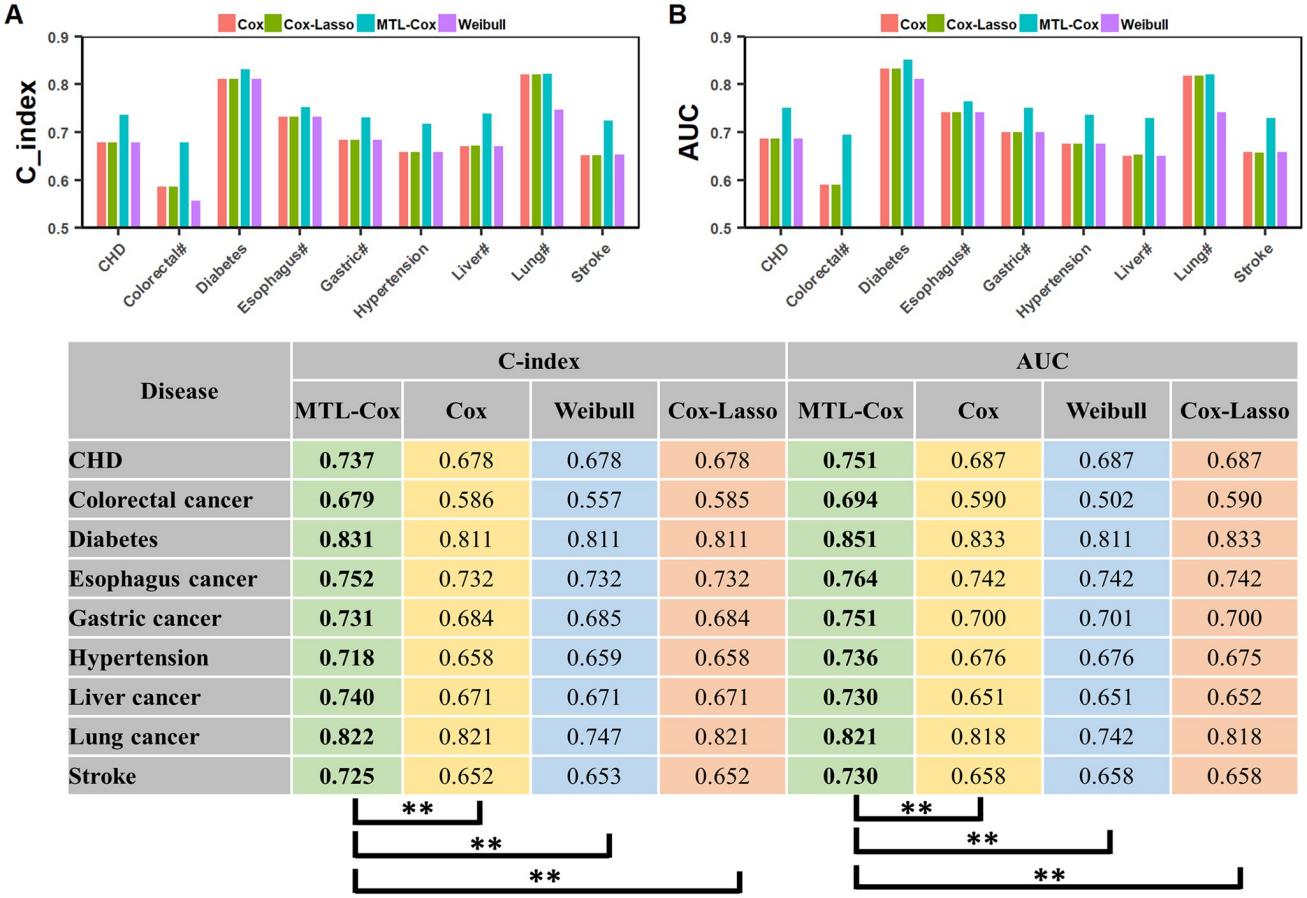
Performances of MTL-Cox and competing methods were evaluated using C-index and AUC. Notes: ‘#’ stands for the word “cancer”, which facilitates the layout of the x-axis labels. ‘*’ denotes *p* < 0.05, ‘**’ denotes *p* < 0.01, and ‘***’ denotes *p* < 0.001.

**Fig 9:**
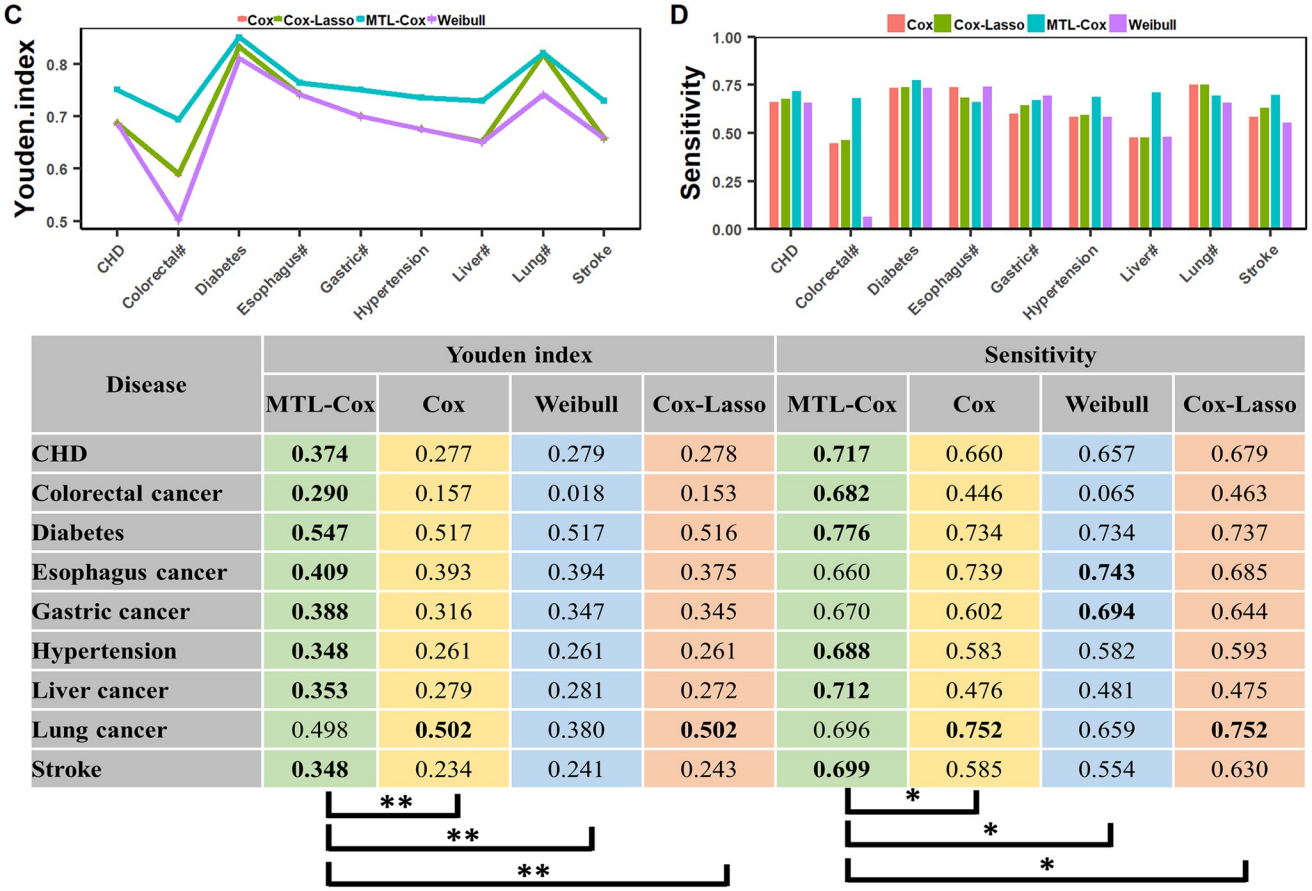
Performances of MTL-Cox and competing methods were evaluated using Youden index and Sensitivity. Notes: ‘#’ stands for the word “cancer”, which facilitates the layout of the x-axis labels. ‘*’ denotes *p* < 0.05, ‘**’ denotes *p* < 0.01, and ‘***’ denotes *p* < 0.001.

**Fig 10:**
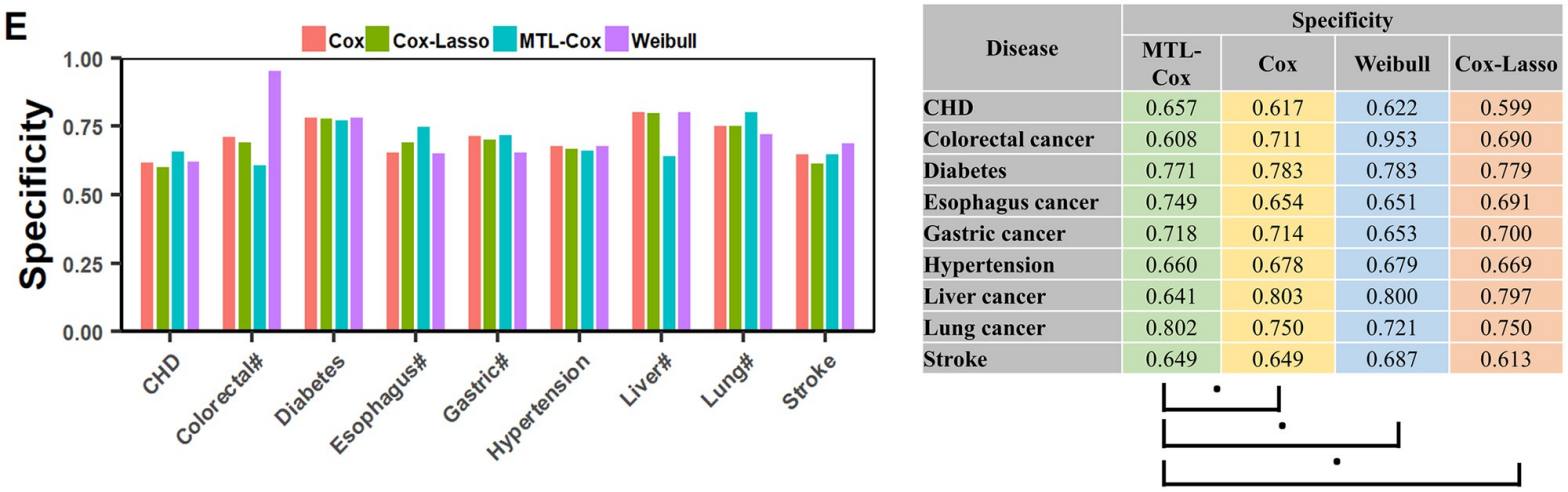
Performances of MTL-Cox and competing methods were evaluated using Specificity. Notes: ‘#’ stands for the word “cancer”, which facilitates the layout of the x-axis labels. ‘.’ denotes *p* > 0.05, ‘*’ denotes *p* < 0.05, ‘**’ denotes *p* < 0.01, and ‘***’ denotes *p* < 0.001.

Experimental results are depicted in Figs 8, 9 and 10, with further details recorded in Supplementary file S14 Table. Our MTL-Cox model outperformed competing methods, demonstrating superior performance in terms of C-index and AUC (*p* < 0.01), as well as sensitivity (*p* < 0.05). Although there was no statistically significant difference based on specificity, it is worth noting that a trade-off exists between sensitivity (missed rates) and specificity (misdiagnosis rates). Therefore, we used the Youden index as a measure to balance sensitivity and specificity when evaluating the overall diagnostic test performance. The results presented in Fig 9 indicate that our MTL-Cox model outperforms competing methods in terms of Youden index (*p* < 0.01).

**Fig 11:**
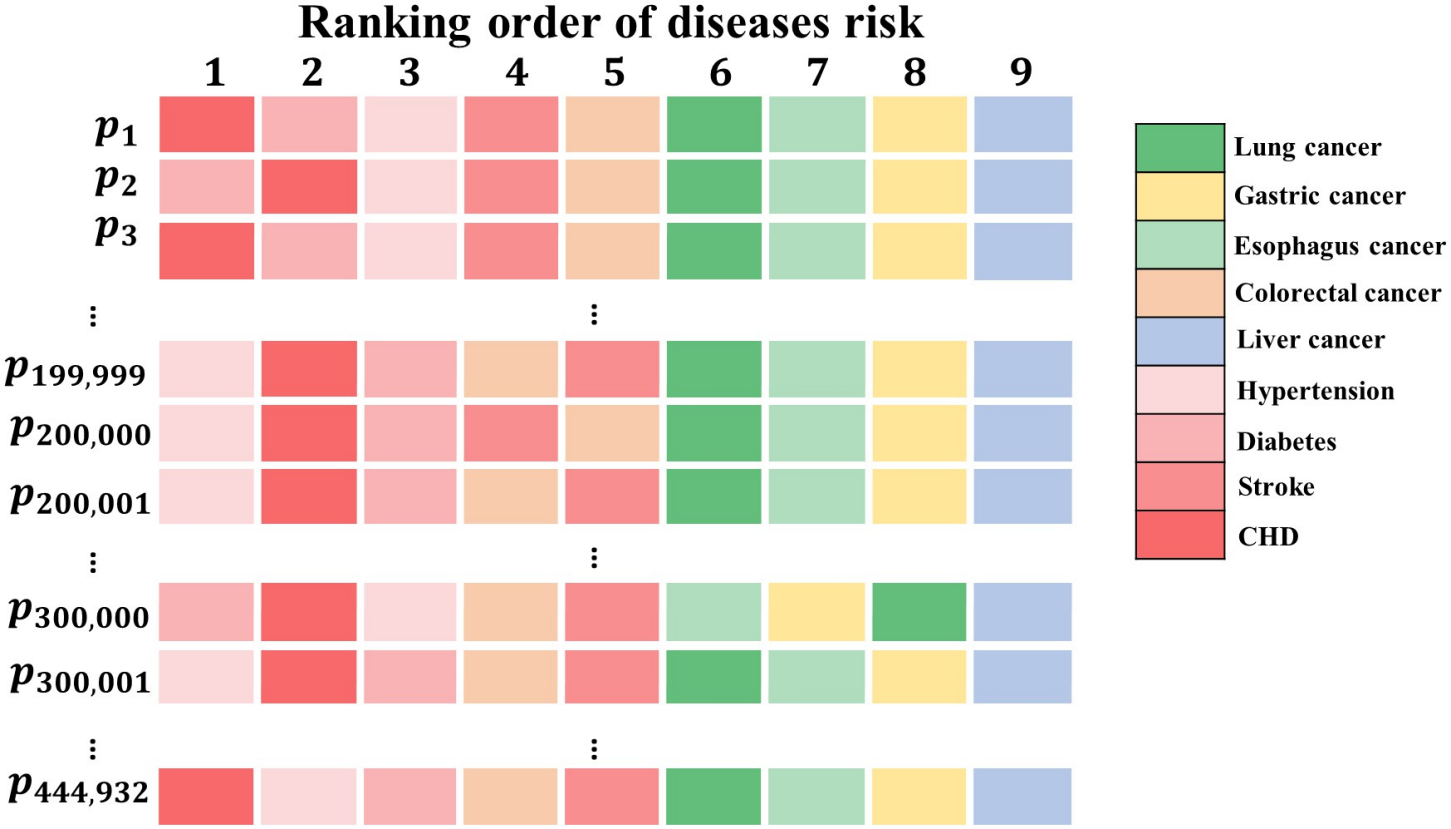
Personalized absolute risk ranking of nine chronic diseases in the fifth year. *p*_*i*_ denotes the *i*–*th* subjects.

**Fig 12:**
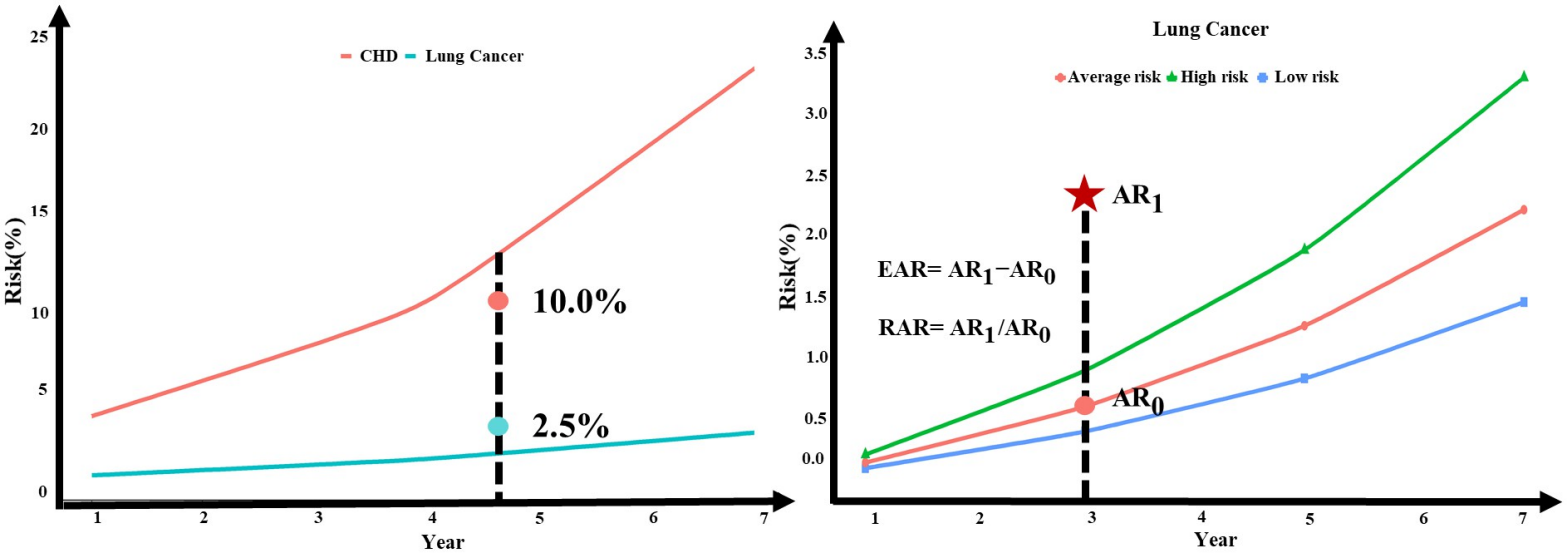
**(A) Illustration of Absolute and Basis Risk for a Population**: The red line in the figure on the left represents the average basis risk for coronary heart disease (CHD), while the green line in the figure on the left represents the average basis risk for lung cancer. The subject’s absolute risk for CHD is approximately 10.0%, and their absolute risk for lung cancer is 2.5%. **(B) Personalized Risk Level Prediction Example**: In the third year, *AR*_1_ represents the subject’s absolute risk of lung cancer, while *AR*_0_ is the basis average risk of lung cancer in the same year. The excess absolute risk is denoted by EAR, and the relative absolute risk is RAR.

As MTL-Cox incorporates the Cox model, it is possible to determine the absolute risk of individual developing diseases using Eq (6). The probability of each subject developing nine chronic diseases in the 1st, 3rd, 5th, and 7th year was calculated and presented in Supplementary file S4, S5 and S6 Tables. For example, using the 5th year, we ranked the absolute risk of the nine chronic diseases for each subject, which is shown in Supplementary file S13 Table and visualized in Fig 11. However, solely relying on absolute risk to judge a subject’s risk level is not enough. For instance, in Fig 12A, the subject’s risk of coronary heart disease is approximately 10.0%, while the risk of lung cancer is 2.5% as per the model. Although the numerical risk level of coronary heart disease is higher than that of lung cancer, the risk of coronary heart disease is still lower than the population’s baseline risk, and it does not indicate the risk of a high-risk individual with coronary heart disease. On the other hand, the risk of lung cancer is higher than the population’s baseline risk and refers to the risk of a high-risk individual with lung cancer. Therefore, mapping the absolute risk to the population’s baseline risk is necessary to better measure the relative absolute risk and excess absolute risk of each subject.

**Fig 13:**
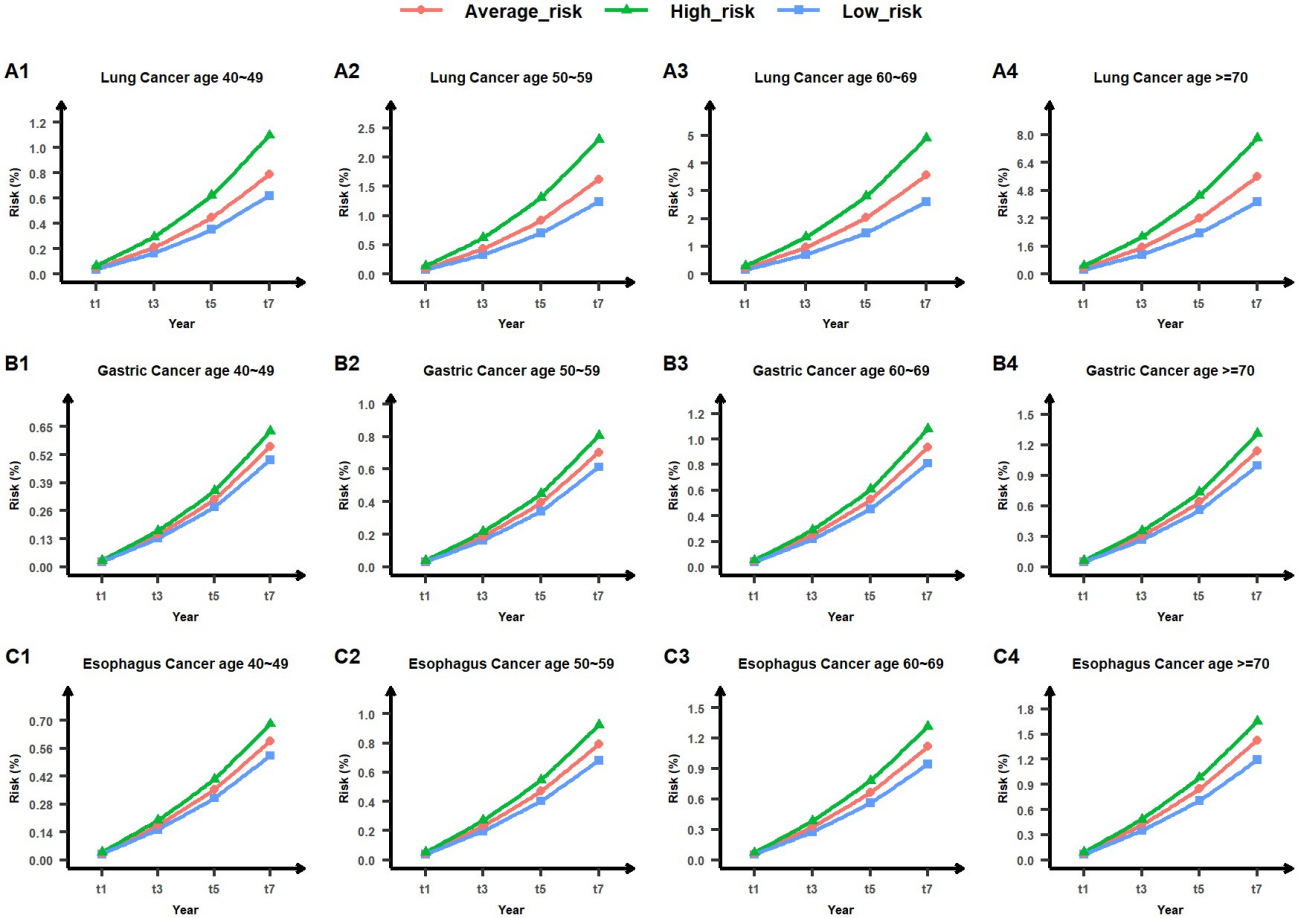
Low-risk, average-risk, and high-risk levels for lung cancer, gastric cancer, and esophagus cancer across four age groups (40∼49, 50∼59, 60∼69, and ≥70). The horizontal axis denotes the timeline in years, while the vertical axis represents the risk value measured in percentage. The area below the blue line indicates low risk, the area between the blue and green lines denotes average risk, and the area above the green line signifies high risk. Comprehensive outcomes are provided in Supplementary file S3 Table.

**Fig 14:**
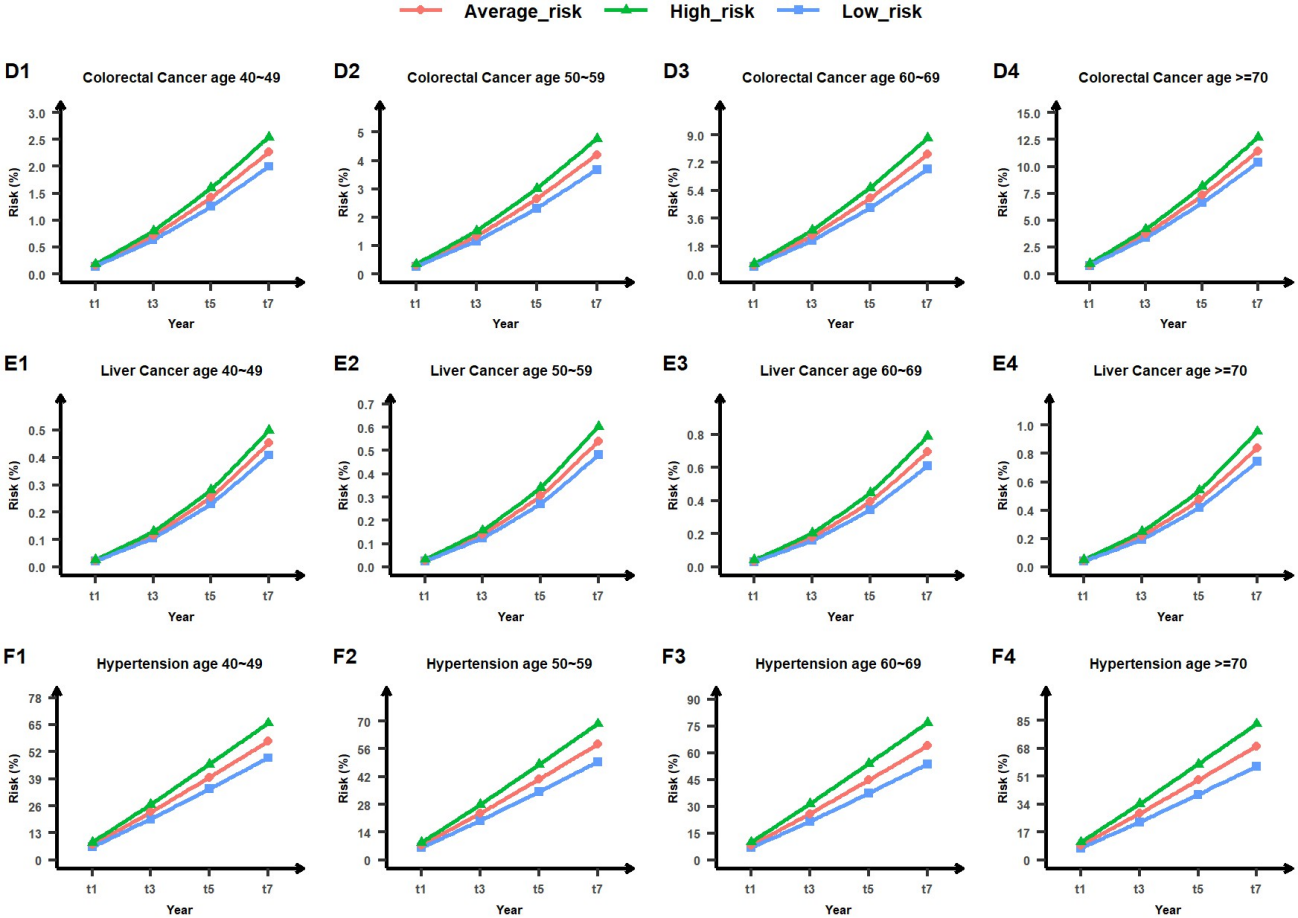
Low-risk, average-risk, and high-risk levels for colorectal cancer, liver cancer, and hypertension across four age groups (40∼49, 50∼59, 60∼69, and ≥70). The horizontal axis denotes the timeline in years, while the vertical axis represents the risk value measured in percentage. The area below the blue line indicates low risk, the area between the blue and green lines denotes average risk, and the area above the green line signifies high risk. Comprehensive outcomes are provided in Supplementary file S3 Table.

**Fig 15:**
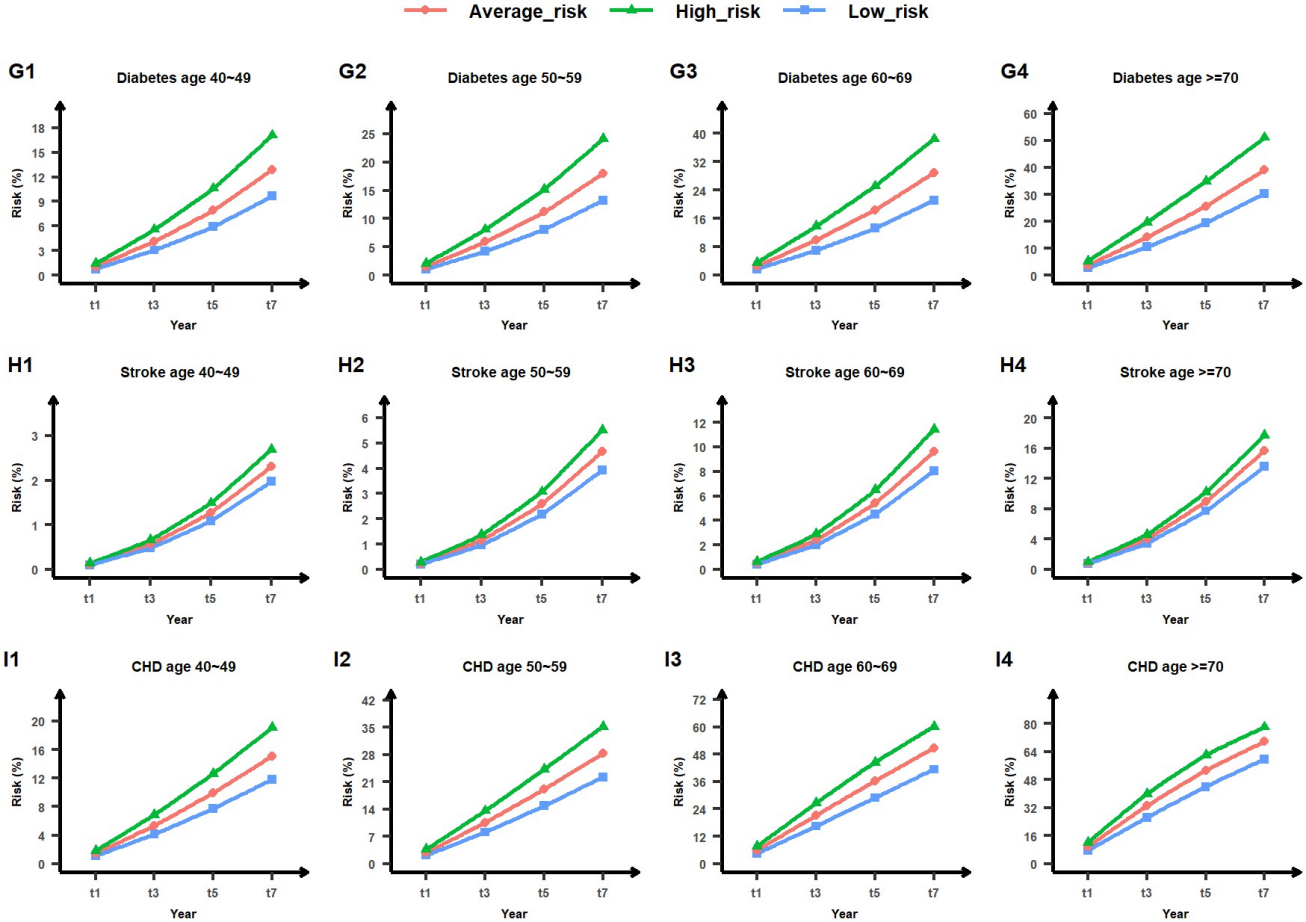
Low-risk, average-risk, and high-risk levels for diabetes, stroke, and coronary heart disease (CHD) across four age groups (40∼49, 50∼59, 60∼69, and ≥70). The horizontal axis denotes the timeline in years, while the vertical axis represents the risk value measured in percentage. The area below the blue line indicates low risk, the area between the blue and green lines denotes average risk, and the area above the green line signifies high risk. Comprehensive outcomes are provided in Supplementary file S3 Table.

We initially categorized the population basis risk output from the MTL-Cox model into three categories: low-risk, average-risk, and high-risk. The absolute risk of each subject was then sorted into the 33%, 50%, and 66% quantiles to establish the upper bound value of the low-risk group, the median value of the average-risk group, and the lower value of the high-risk group. Next, we created population basis lines for low-risk, average-risk, and high-risk groups for nine chronic diseases, as shown in Figs 13, 14 and 15. Finally, we illustrated the risk of lung cancer for a sample subject after one year and three years. As depicted in Fig 12B, this subject’s risk of developing lung cancer after one year and three years was greater than that of the high-risk population. Additionally, their risk of lung cancer after three years was 4.02 times higher than the population’s average absolute risk, with an excess absolute risk of 1.79340% after three years. Therefore, this subject is considered a high-risk individual for lung cancer, and this finding can help remind both the subject and their physician to take appropriate treatment measures as soon as possible. Relative absolute risk for all subjects can be found in Supplementary files S7, S8 and S9 Tables. Additionally, the excess absolute risk for all subjects can be found in Supplementary files S10, S11 and S12 Tables.

## Discussion

In the experimental results section, we present the evaluation of the MTL-Cox model from the perspective of five quantitative evaluation metrics. Our experimental results show that the MTL-Cox model outperforms other competing single-task learning methods in terms of concordance index, AUC, sensitivity, and the Youden index. However, there was no clear advantage in terms of specificity. Regarding the concordance index, we observe that MTL-Cox performed with the highest values in all diseases, indicating its superior precision. Additionally, the higher AUC value of MTL-Cox compared to single-task learning methods suggests its better performance in discriminating between diseased and healthy populations. We also observe that the MTL-Cox model outperforms other methods in terms of sensitivity, which is crucial in detecting disease in patients and reducing missed diagnoses. With regards to specificity, we noted that there was no significant difference between the MTL-Cox model and other single-task learning methods on the UK Biobank. This is because sensitivity and specificity are inversely related when the population prevalence remains constant [68]. However, the Youden index of the MTL-Cox model was significantly higher than competing methods, indicating its advantage in precision measurement when considering both the missed diagnosis rate and the misdiagnosis rate.

Moreover, we take gastric cancer prediction in the UK Biobank dataset as an example to demonstrate the accuracy of the MTL-Cox model. We predict the risk probability of developing gastric cancer in the fifth year and convert it into binary classification (0 for not developing gastric cancer, 1 for developing) using the cut-off value obtained from the AUC. Among those who developed gastric cancer, 108 individuals were predicted accurately by the MTL model while other competing methods failed to do so. This accurate prediction is attributed to the multitask learning framework employed in the MTL-Cox model. The multitask learning framework of the model allows for shared training information and knowledge, leading to an effective increase in sample size and improving the generalization ability of the model [27]. The advantages of multitask learning include data amplification, eavesdropping, attention focusing, representation bias, and overfitting prevention [27]. Data amplification refers to an effective increase in sample size due to extra information in the training signals of related tasks. Eavesdropping allows a task to learn features that are difficult for another task to learn. Attention focusing helps the model distinguish between relevant and irrelevant features. Representation bias prefers to learn a class of models that also emphasizes other tasks, which helps the model demonstrate generalization to new tasks. Overfitting prevention is achieved by the shared module in multitask learning, which takes into account all tasks and avoids overfitting to a single task’s training set. However, the specific mechanism of the superior performance of the multitask learning framework cannot be proved theoretically, and future work is needed to analyze the internal mechanism of the MTL-Cox model.

Furthermore, in terms of feature selection, our goal is to predict diseases using a precise and minimal feature set, considering both the clinical practicality and computational efficiency of the MTL-Cox model in high-dimensional data. To achieve this, we conduct feature selection based on a single disease, as each chronic disease has specific biological characteristics and pathological mechanisms. This approach helps us more accurately screen variables related to the target disease. However, considering the possibility of missing variables, we take into account the correlation between chronic diseases and use the union of all variables preliminarily screened from chronic diseases as the input for the MTL-Cox model. It is worth noting that, as described in the paper, we have incorporated a regularization term, namely the *L*_2,1_ norm, into the MTL-Cox model. This regularization term takes into account the relationships between multiple chronic diseases for feature selection while conducting feature selection for multiple diseases, rather than selecting features based on a single disease as in single-task learning. This also highlights the advantages of the MTL-Cox model in feature selection, as it can simultaneously consider the correlations and differences between multiple diseases, and find the optimal feature sets with predictive power for multiple diseases. However, this feature selection strategy is not perfect for high-dimensional data. Therefore, in future work, researchers can further improve the MTL-Cox model to enable rapid variable selection and prediction in high-dimensional data.

## Conclusion

Currently, chronic disease prevention and control are limited, and the incidence and mortality rates of chronic diseases remain high and are expected to keep rising [69–71]. This is due to the unclear etiology and pathogenesis of most chronic diseases, as well as the poor treatment effect. Therefore, preventing the occurrence of chronic diseases is of practical significance. In this study, we designed a risk prediction model for nine chronic diseases based on the MTL-Cox model using UK Biobank data. We also validated the MTL-Cox model’s effectiveness using the Weihai physical examination database. The MTL-Cox model can handle survival data with the right censoring by embedding the Cox proportional hazards model and consider the task-relatedness of chronic diseases by using multitask learning. Furthermore, we applied five performance metrics to evaluate the survival analysis model: C-index, AUC, specificity, sensitivity, and Youden index, to measure the model’s prediction performance. Compared with other models, the MTL-Cox model leveraged more data from different learning tasks than single-task learning, leading to better knowledge sharing between tasks, better performance of each task, and a low risk of overfitting for each task [28]. As a result, the MTL-Cox model was found to be a reliable early diagnosis method for clinical medicine. Additionally, we calculated the absolute risk of subjects from different years based on the MTL-Cox model and ranked the risk. Subsequently, the risks of the nine chronic diseases in the UK Biobank were graded to achieve hierarchical early warning. Finally, to accurately quantify the risk of subjects, we mapped individuals’ absolute risk to the population basis risk, which reflected the relative absolute risk and excess absolute risk. The MTL-Cox model can guide the lives of individuals based on input features, especially in high-risk groups, thereby reducing the risk of chronic diseases. Overall, this study contributes to the prevention and early detection of chronic diseases, which is of great importance for public health.

## Future work

There are several limitations to this study that will be addressed in future research. Firstly, the age range of the UK Biobank dataset used in this paper was limited to individuals aged between 37 to 73 years, which may have introduced selection bias as chronic diseases are more prevalent in the elderly population (https://www.cdc.gov/nchs/health_policy/adult_chronic_conditions.htm. Accessed on Spe. 17^*th*^, 2022.). Secondly, future studies will consider incorporating multimodal data related to chronic diseases to improve the generalizability of the model. Thirdly, developing feature selection methods of high-dimensional data for the MTL-Cox model will be a subject of future work.

## Supporting information

S1 Table Details of demographic and clinical features. The details of demographic and clinical features, including the names of features, features type, and the description of features.
(XLSX)

S2 Table Baseline information for nine chronic diseases. The information for the nine chronic diseases we studied on the UK Biobank dataset.
(XLSX)

S3 Table Low risk, average risk, and high risk of nine chronic diseases. The cut-off level of low risk, average risk, and high risk for nine chronic diseases.
(XLSX)

S4 Table Personalized predicted probabilities of lung cancer, gastric cancer, and esophagus cancer_Absolute Risk. The absolute risk of each subject developing lung cancer, gastric cancer, and esophagus cancer in the 1-*th*, 3-*th*, 5-*th*, and 7-*th* year, respectively.
(XLSX)

S5 Table Personalized predicted probabilities of colorectal cancer, liver cancer, and hypertension_Absolute Risk. The absolute risk of each subject developing colorectal cancer, liver cancer, and hypertension in the 1-*th*, 3-*th*, 5-*th*, and 7-*th* year, respectively.
(XLSX)

S6 Table Personalized predicted probabilities of diabetes, stroke, and CHD_Absolute Risk. The absolute risk of each subject developing diabetes, stroke, and CHD in the 1-*th*, 3-*th*, 5-*th*, and 7-*th* year, respectively.
(XLSX)

S7 Table Personalized predicted probabilities of lung cancer, gastric cancer, and esophagus cancer_Relative Absolute Risk. The relative absolute risk of each subject developing lung cancer, gastric cancer, and esophagus cancer in the 1-*th*, 3-*th*, 5-*th*, and 7-*th* year, respectively, which reflects that the absolute risk of each individual is a multiple of the average absolute risk of the population.
(XLSX)

S8 Table Personalized predicted probabilities of colorectal cancer, liver cancer, and hypertension_Relative Absolute Risk. The relative absolute risk of each subject developing colorectal cancer, liver cancer, and hypertension in the 1-*th*, 3-*th*, 5-*th*, and 7-*th* year, respectively, which reflects that the absolute risk of each individual is a multiple of the average absolute risk of the population.
(XLSX)

S9 Table Personalized predicted probabilities of diabetes, stroke, and CHD_Relative Absolute Risk. The relative absolute risk of each subject developing diabetes, stroke, and CHD in the 1-*th*, 3-*th*, 5-*th*, and 7-*th* year, respectively, which reflects that the absolute risk of each individual is a multiple of the average absolute risk of the population.
(XLSX)

S10 Table Personalized predicted probabilities of lung cancer, gastric cancer, and esophagus cancer_Excess Absolute Risk. The excess absolute risk of each subject developing lung cancer, gastric cancer, and esophagus cancer in the 1-*th*, 3-*th*, 5-*th*, and 7-*th* year, respectively, which reflects the difference between the absolute risk of each individual and the average absolute risk of everyone.
(XLSX)

S11 Table Personalized predicted probabilities of colorectal cancer, liver cancer, and hypertension_Excess Absolute Risk. The excess absolute risk of each subject developing colorectal cancer, liver cancer, and hypertension in the 1-*th*, 3-*th*, 5-*th*, and 7-*th* year, respectively, which reflects the difference between the absolute risk of each individual and the average absolute risk of everyone.
(XLSX)

S12 Table Personalized predicted probabilities of diabetes, stroke, and CHD_Excess Absolute Risk. The excess absolute risk of each subject developing diabetes, stroke, and CHD in the 1-*th*, 3-*th*, 5-*th*, and 7-*th* year, respectively, which reflects the difference between the absolute risk of each individual and the average absolute risk of everyone.
(XLSX)

S13 Table Absolute risk ranking of nine chronic diseases in the fifth year for each subject. Taking the fifth year as an example, the absolute risk of nine chronic diseases per subject was ranked. The values in the table are absolute risk, and the darker the color, the higher the risk.
(XLSX)

S14 Table Five evaluation metrics for four models. The experimental results between different methods are reported in this table.
(XLSX)

S15 Table Categorical feature coding details. The details of categorical features coding.
(XLSX)

S16 Table The features details of the Weihai physical examination dataset. The details of features on the Weihai physical examination dataset, including the names of features, the description of features, and features initially selected for each target disease.
(XLSX)

S17 Table Baseline information for five cancers of the Weihai physical examination dataset. The information for the nine chronic diseases we studied on the Weihai physical examination dataset.
(XLSX)

S1 File Validating the MTL-Cox model framework in the Weihai physical examination dataset. The details of materials and experiment results are based on the Weihai physical examination dataset.
(DOCX)
