# Supplementary material for: Personalized prediction for multiple chronic diseases by developing the multi-task Cox learning model
Source: PLoS Comput Biol. 2023 Sep 21;19(9):e1011396. doi: 10.1371/journal.pcbi.1011396 (PMC10569718; doi:10.1371/journal.pcbi.1011396)
Supplement: S1 File — The details of materials and experiment results are based on the Weihai physical examination dataset. (DOCX) [file pcbi.1011396.s018.docx]

**S1 File. Validating the MTL-Cox model framework in the Weihai physical examination dataset. The details of materials and experiment results based on the Weihai physical examination dataset**

# Materials

## Ethics statement

This study was approved by the Ethics Committee of Weihai Municipal Hospital affiliated to Shandong University. Our study was based on the health checkup population, before conducting health checkup, all participants were required to choose whether they agree to contribute their data for clinical research through a questionnaire. All participants included in this study gave their consent to participate in this study.

## Datasets

The Weihai physical examination dataset is a screening dataset collected by the Health Management Department of Weihai City Hospital, which is the largest hospital in Weihai City. Weihai City Hospital is a modern first-class comprehensive hospital equipped with medical, therapeutic, teaching, research, emergency care, and preventive medicine. It is also the medical center of Weihai City. The Health Management Department of Weihai City Hospital was established in 2006. In June 2019, it successfully passed the review of the National "Health Management Building and Science and Technology Innovation Center" project, becoming the first health management demonstration base in the Weihai area, marking a leading position in the industry in terms of disciplines, service management, information technology construction, quality control, cultural construction, and innovative characteristics.

The physical examination cohort of Weihai City Hospital, located in Shandong Province, China, has a total of 866,827 records, including 225,135 people with demographic information, diseases, medications, and laboratory tests. The follow-up time was from 2013 to 2021, with an average follow-up time of 6.2 years and a maximum follow-up time of 9 years.

In the Weihai physical examination cohort, we focused on five chronic diseases, namely lung cancer, gastric cancer, esophagus cancer, colorectal cancer, and liver cancer. The participant's follow-up period commenced upon their inclusion in the cohort and continued until December 31, 2021 or until a cancer diagnosis was made. We excluded participants who had a prior diagnosis of the target disease before joining the cohort. Additionally, individuals under the age of 18 were excluded, leaving a participant pool ranging in age from 18 to 93 years old. Follow-ups were deemed censored if events of interest were not detected for various reasons.

## Feature selection

The variable selection process was consistent with the UK Biobank dataset. A total of 1624 features were considered, comprising 1548 disease features, 76 demographic features and laboratory test features. The selection was made using the combination of mono-factor analysis initial screening, expert knowledge, and forward regression screening. Ultimately, a total of 32 features as input features of MTL-Cox were selected out of the five cancers. The details of selected features are listed in the Supplementary file S10_Table, and baseline information is presented in the Supplementary file S11_Table.

# Results

Fig. 1 shows the experimental results based on the Weihai physical examination dataset. The MTL-Cox model outperforms competing methods in metrics of C-index (p < 0.05), AUC (p < 0.05), and sensitivity (p < 0.05). However, there is no advantage in terms of specificity. Since there is a trade-off between sensitivity and specificity, the Youden index is used to balance both metrics and evaluate the overall ability of the diagnostic test. In this case, the results show that the MTL-Cox model outperforms competing methods based on the Youden index (p < 0.05).

| 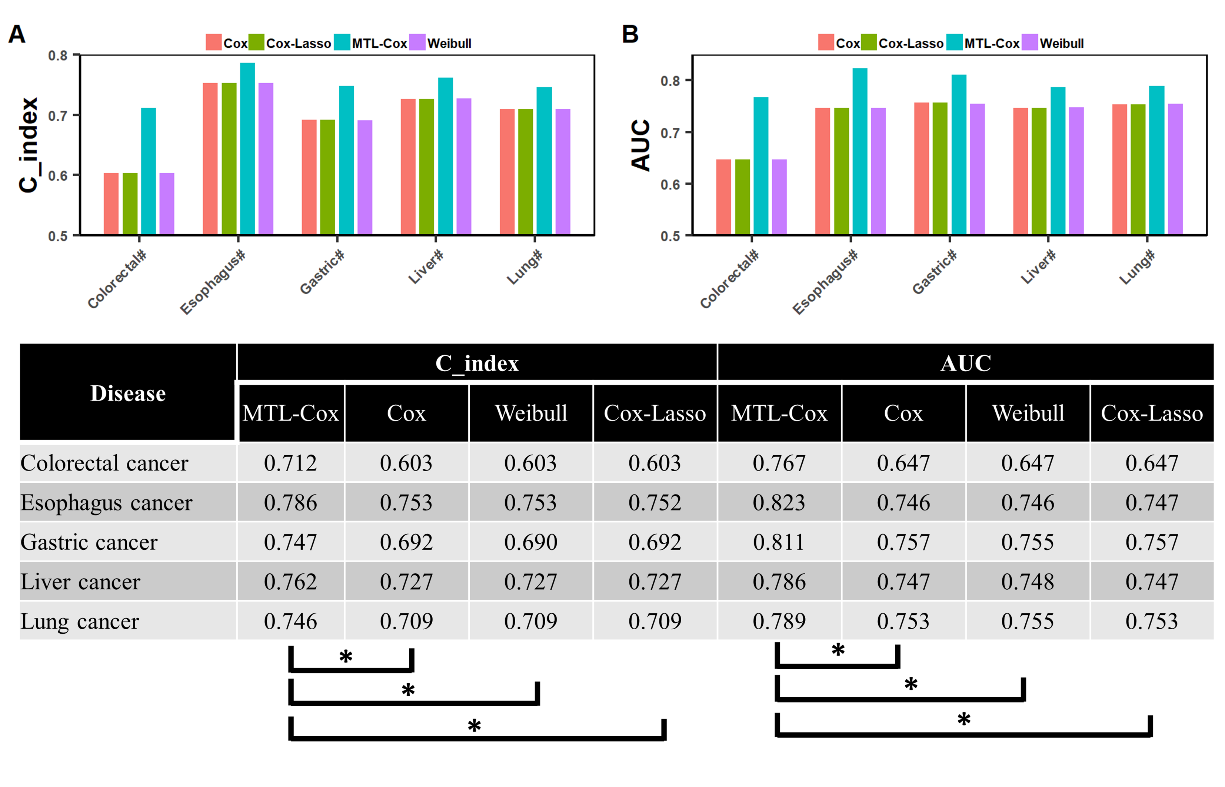 |
| --- |
| 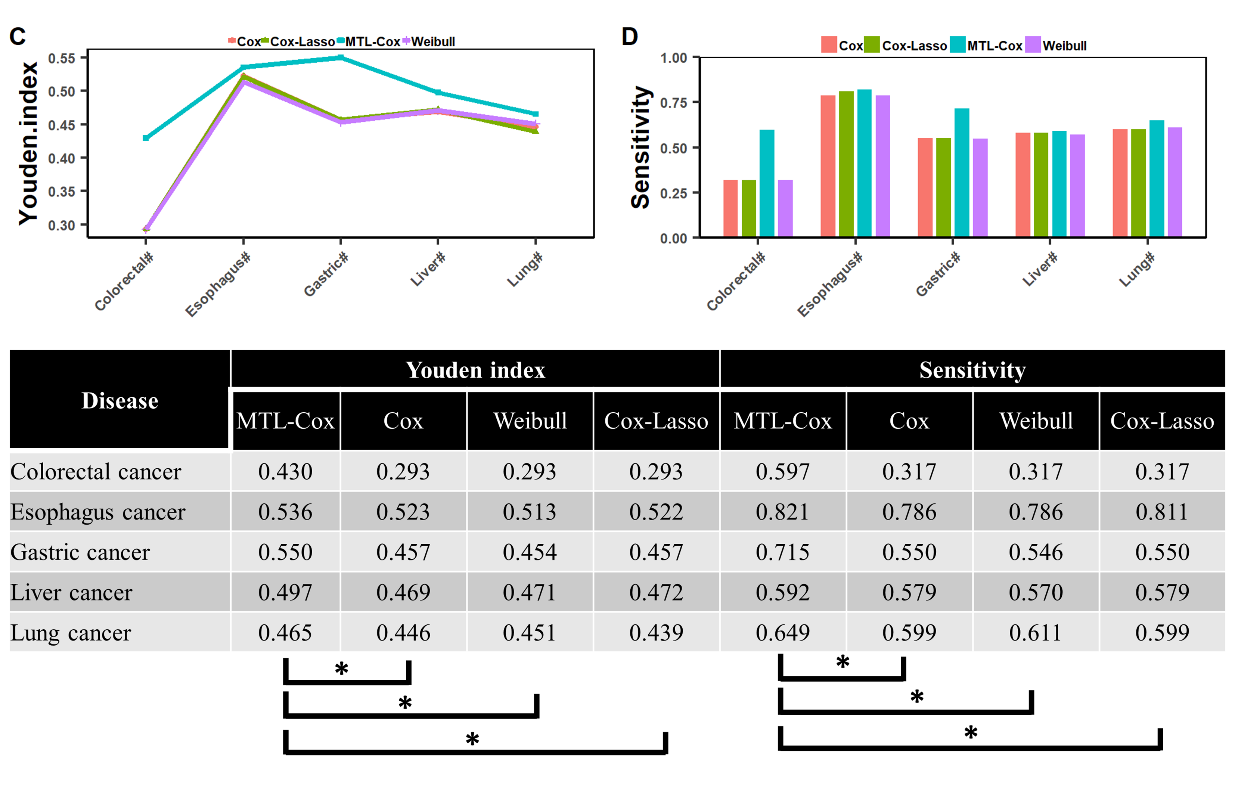 |
| 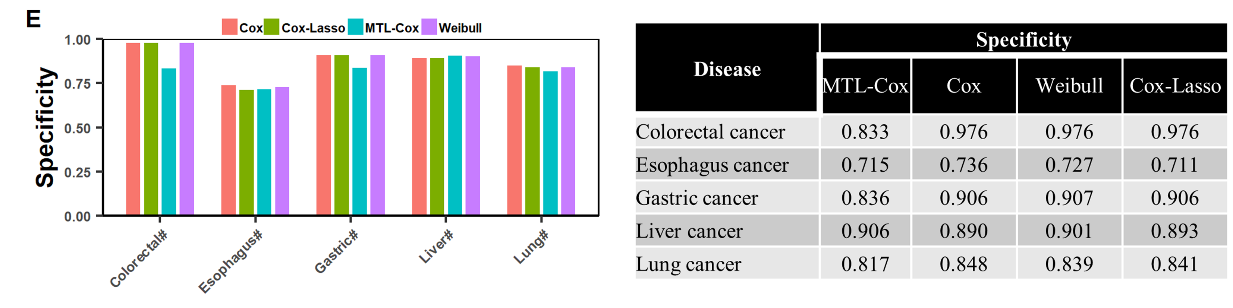 |
| Fig 1. Performances based on C-index, AUC, sensitivity, specificity, and the Youden index for MTL-Cox and competing methods. Notes: ’#’ stands for the word ”cancer”, which facilitates the layout of the x-axis labels. ’*’ denotes p < 0.05. |
